# Crystal structures of 2,3,7,8,12,13,17,18-octa­bromo-5,10,15,20-tetra­kis­(penta­fluoro­phen­yl)porphyrin as the chloro­form monosolvate and tetra­hydro­furan monosolvate

**DOI:** 10.1107/S2056989020000432

**Published:** 2020-01-17

**Authors:** Christopher J. Kingsbury, Keith J. Flanagan, Marc Kielmann, Brendan Twamley, Mathias O. Senge

**Affiliations:** a School of Chemistry, Trinity Biomedical Science Institute, 152–160 Pearse Street, Trinity College Dublin, The University of Dublin, Dublin 2, Ireland

**Keywords:** crystal structure, symmetrical porphyrins, non-planar porphyrins, halogenated porphyrins, solvent inter­actions

## Abstract

The crystal structures of the title compounds, two solvates (CHCl_3_ and THF) of a symmetric and highly substituted porphyrin, C_44_H_2_Br_8_F_20_N_4_ or OBrTPFPP, are described. These structures each feature a non-planar porphyrin ring, exhibiting a similar conformation of the strained ring independent of solvent identity. These distorted porphyrins are able to form hydrogen bonds and sub-van der Waals halogen inter­actions with enclathrated solvent; supra­molecular inter­actions of proximal macrocycles are additionally affected by solvent choice. The crystal studied for compound **1·CHCl_3_** was refined as an inversion twin. One penta­fluoro­phenyl group was modelled as disordered over two sites [occupancy ratio = 0.462 (7):0.538 (7)]. The chloro­form solvate was also modelled as disordered over two orientations [occupancy ratio = 0.882 (7): 0.118 (7).

## Abstract 2

The crystal structures of the title compounds, two solvates (CHCl_3_ and THF) of a symmetric and highly substituted porphyrin, OBrTPFPP, are described. These structures each feature a non-planar porphyrin ring, exhibiting a similar conformation of the strained ring independent of solvent identity. These distorted porphyrins are able to form hydrogen bonds and sub-van der Waals halogen inter­actions with enclathrated solvent; supra­molecular inter­actions of proximal macrocycles are additionally affected by solvent choice.

## Chemical context   

Highly substituted porphyrins are a subclass of porphyrin compounds where the *meso* and β positions are substituted with non-H atoms. When large substituents are introduced to the periphery of the porphyrin ring, this tends to overcrowd the macrocycle and induce conformational distortion, increasing with the steric demand (Senge & Kalisch, 1997 ▸; Medforth *et al.*, 1992 ▸). Among the most studied substitution patterns are those with variously functionalized aryl rings at the 5,10,15,20-positions and with halogen, alkane and aryl substituents at the 2,3,7,8,12,13,17,18-positions (Senge, 2000 ▸, 2006 ▸)

There are numerous approaches used to introduce conformational distortion to porphyrins, including coordination of specific metal centers, incorporation of a strapping motif, or decorating the ring with sterically demanding substituents (Schindler *et al.*, 2018 ▸; Senge, 2006 ▸). Recent publications show uses for distorted porphyrins as free-base catalysts and sensors, and these compounds demonstrate unique and tuneable porphyrin inner core inter­actions (Aoki *et al.*, 2019 ▸; Norvaiša *et al.*, 2019 ▸; Kielmann *et al.*, 2019 ▸; Kielmann & Senge, 2019 ▸). For example, non-planar metal-free porphyrins show promise as organocatalysts, acting as hydrogen-bond donors (Kielmann *et al.*, 2019 ▸). Moreover, the porphyrin scaffold is customizable, and the potential for tuneable basicity and catalytic activity based on variable substitution patterns has been explored (Roucan *et al.*, 2017 ▸). The distortion of the porphyrin ring, when compared to the planar parent compound, affects the photophysical and electronic properties of both free-base macrocycles and of derived metal complexes (Parusel *et al.*, 2000 ▸; Gentemann *et al.*, 1994 ▸; Röder *et al.*, 2010 ▸). With this in mind, halogenated porphyrins specifically are of inter­est as ligands in catalytic metal complexes, owing to non-planar conformation, as well as the electron-deficient character of the porphyrin ring (Dolphin *et al.*, 1997 ▸; Henling *et al.*, 1993 ▸).
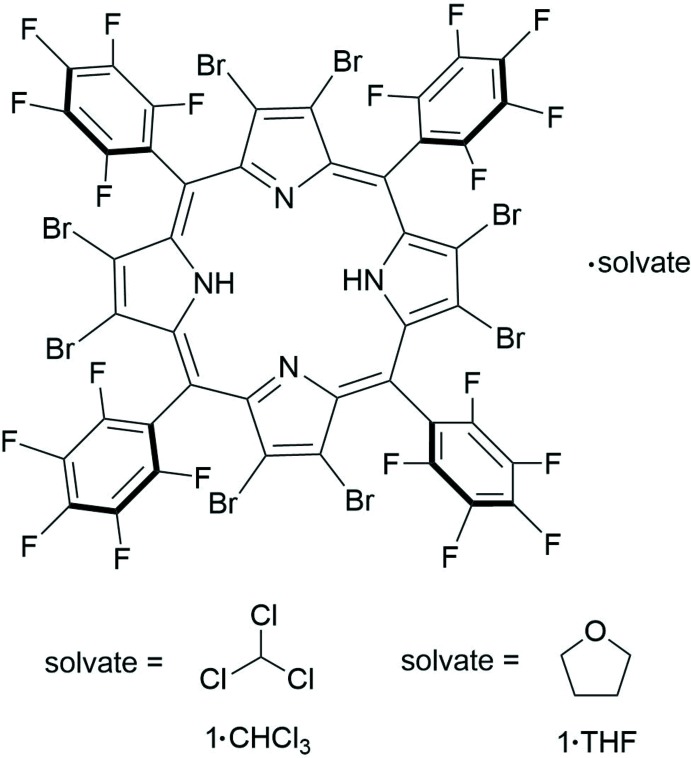


The title compound has been previously characterized as a di­chloro­benzene solvate (Takeuchi *et al.*, 1994 ▸). Structural differences between this literature compound and the structures presented herein arise from inter­molecular inter­actions with chloro­form and THF. Additionally, in the published structure, the solvent could not be adequately modelled. These three structures are compared below.

## Structural commentary   

**Figure 1:**
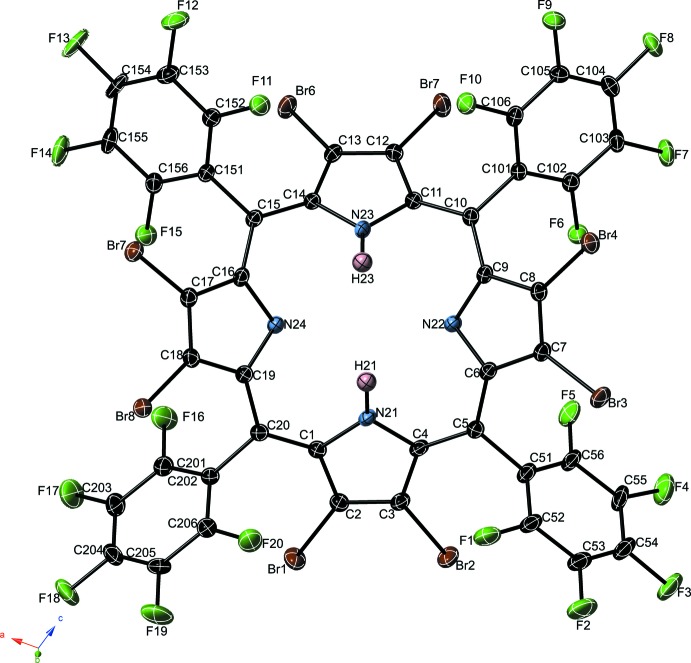
View of the mol­ecular structure of the main residue of **1·CHCl_3_**; displacement ellipsoids are drawn at the 50% probability level. A second disordered orientation of the C151–156 ring and the solvent chloro­form mol­ecule are omitted for clarity.

The crystal structure of the title compound (2,3,7,8,12,13,17,18-octa­bromo-5,10,15,20-tetra­kis­(penta­fluoro­phen­yl)por­phyrin mono­chloro­form solvate, (**1·CHCl­_3_**) shows a single H_2_OBrTPFPP mol­ecule and one chloro­form solvate in the asymmetric unit. This highly substituted porphyrin ring exhibits *peri* inter­actions from the appended bromine atoms crowding with penta­fluoro­phenyl rings, forcing the bromine atoms substanti­ally out of the mean plane of the porphyrin ring at a mean deviation of 2.14 (14) Å. One of these penta­fluoro­phenyl rings is disordered over two positions, related by a co-planar shift; the co-crystallized CHCl_3_ is also disordered over two orientations. A view of the mol­ecular structure of H_2_OBrTPFPP is shown in Fig. 1 ▸.

A second crystal structure of (2,3,7,8,12,13,17,18-octa­bromo-5,10,15,20-tetra­kis­(penta­fluoro­phen­yl)porphyrin mono­tetra­hydro­furan solvate (**1·THF**) displays essentially the same conformation of the macrocycle; differences in the packing of these compounds are discussed below. The Δ24 values, a summation of atomic deviations from mean plane of the macrocycle are similar in **1·CHCl­_3_ and 1·THF**; a view of the skeletal deviations from the mean-plane in the crystal structure of these two compounds is shown in Fig. 4 ▸ *b*.

Normal Structure Decomposition (NSD, Jentzen *et al.*, 1995 ▸; Schindler *et al.*, 2018 ▸) analysis is the standard method of comparing the mode and extent of distortion between porphyrin structures. NSD is the decomposition of the atomic coordinates of a porphyrin into defined in-plane and out-of-plane distortion modes, based on a least-squares fit of the atomic coordinates to the calculated lowest frequency vibrational modes. The porphyrin rings of the title compounds are shown to exhibit significant out-of-plane saddle-type [B_2u_ (min)] distortion in both crystal structures reported here. This saddling distortion is a direct result of large substituents appended to the porphyrin ring – the *saddle* distortion allevi­ates steric demand by removing the restraint of co-planarity from the Br and aryl groups. Slight isotropic contraction, or *bre* mode distortion, of this porphyrin ring when projected into the *xy-*plane [A_1g_ (min)] is an effect of the large skewing, or pyrrole tilt – the reported Cl_8_TPFPP and F_8_TPFPP porphyrins do not show this A_1g_ contraction with similar bond distances reported, as shown in Table 3 ▸ and Fig. 5 ▸.

## Supra­molecular features   

**Figure 2:**
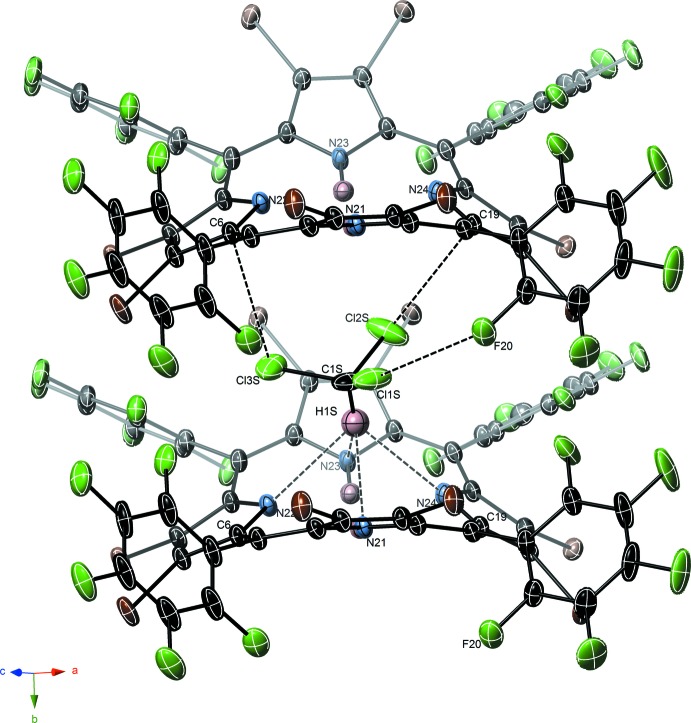
Inter­actions between **1·CHCl_3_** and enclathrated chloro­form solvate, showing proximal Cl⋯F and C—H⋯N inter­actions, indicated by dashed lines. Only the major occupancy component of the chloroform solvent is shown.

In **1·CHCl_3_**, solvent chloro­form mol­ecules are nestled between two adjacent porphyrin rings and disordered over two similar orientations. In the dominant orientation, CHCl_3_ mol­ecules show weak C—H⋯N inter­actions (≃2.7 Å H⋯N) to imine pyrrole rings of one porphyrin, and Cl⋯π inter­actions (≃3.3 Å) as well as Cl⋯F contact (≃3.0 Å) to an adjacent porphyrin (Table 1 ▸). These inter­actions are shown in Fig. 2 ▸.

**Figure 3:**
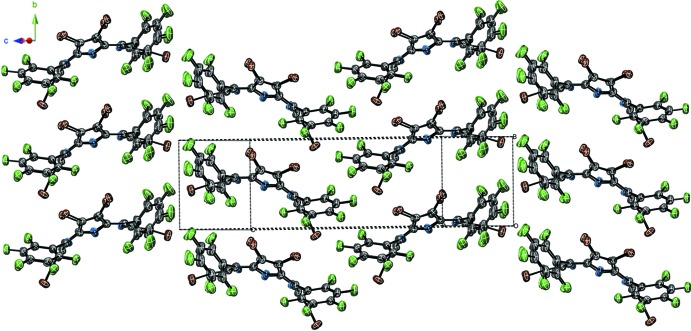
Packing diagram showing the inter­digital arrangement of adjacent stacks of **1·CHCl_3_**. The chloroform solvent is not shown.

This solvent-mediated supra­molecular motif serves to arrange the porphyrin rings directly above and below one another, in an approximately face-to-face arrangement. As a result of this arrangement, the porphyrin mol­ecules form stacks which extend along the *b*-axis direction. The adjacent stacks of porphyrin units in the *ac* direction inter­digitate with one another, as shown in Fig. 3 ▸.

In **1·THF**, the central core of the porphyrin displays traditional hydrogen bonding (Table 2 ▸) from one pyrrole group to the THF oxygen atom [N⋯O 2.849 (6) Å], with a longer distance to the other available pyrrole N—H group (N⋯O = 3.8 Å). The THF solvate is not observed to form similar bimodal intra­molecular inter­actions as the chloro­form solvate, and porphyrin mol­ecules do not form the infinite stacking arrangements seen in **1·CHCl_3_**. The porphyrin mol­ecules display multiple halogen–halogen inter­actions from the bromine and fluorine atoms in both structures.

## Database survey   

**Figure 4:**
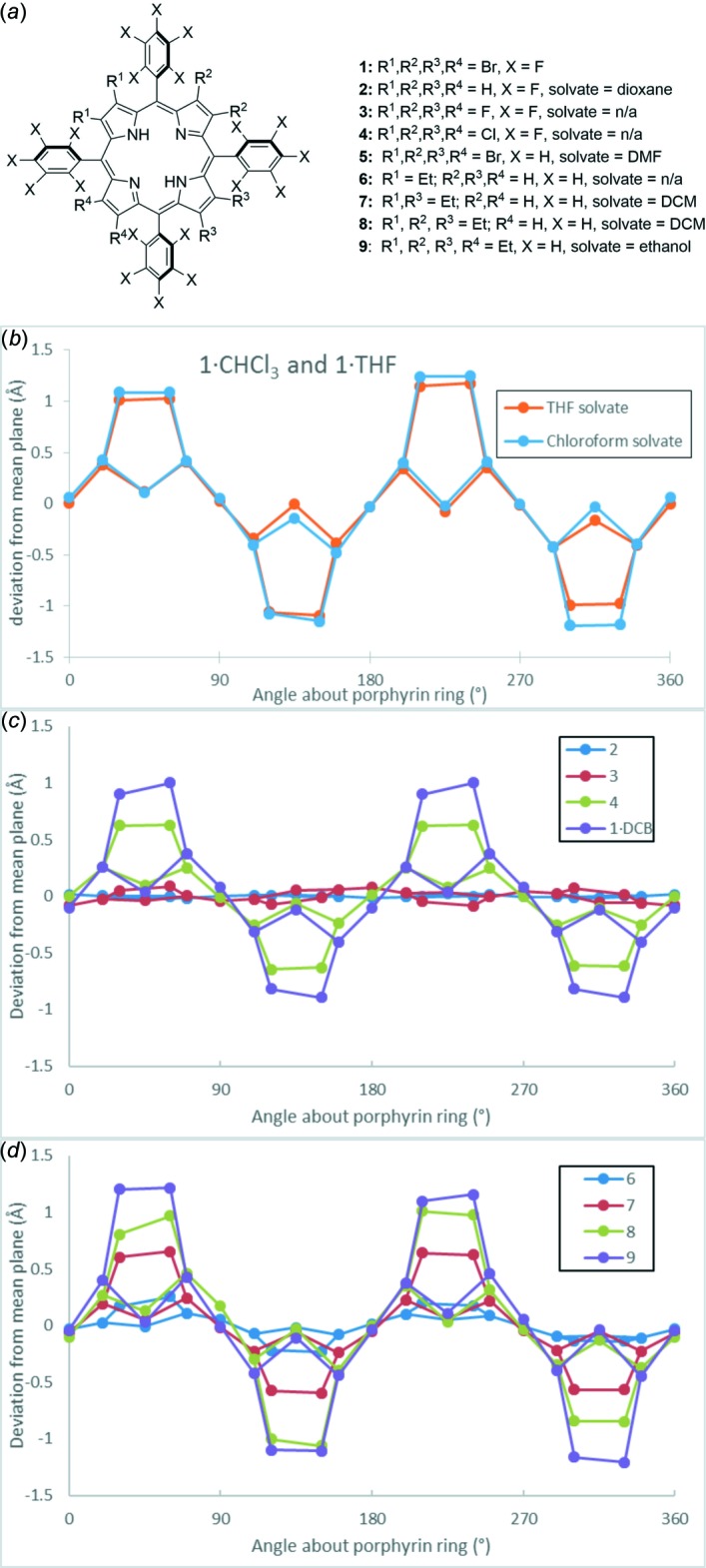
(*a*) Diagram of porphyrin mol­ecules which are used for comparison within this text. (*b*) Comparative mean-plane deviations of compounds **1·CHCl_3_** and **1·THF**. (*c*) Comparative mean-plane deviations for atoms within the β-octa-substituted 5,10,15,20-tetra­kis­(penta­fluoro­phen­yl)porphyrin structures H_2_ *X*_8_TPFPP [**2**: *X* = H (HALZUP), **3**: *X* = F (GODYON), **4**: *X* = Cl (ZALHUP), **1·C_6_H_4_Cl_2_**: *X* = Br (ZALJEB)]. (*d*) Comparative mean-plane deviations for atoms within structures with the formula H_2_[Et_*x*_H_8 - *x*_(TPP)]: **6**: *x* = 2 (TATPOT01), **7**: *x* = 4 (TATPUZ01), **8**: *x* = 6 (TATQEK01), **9**: *x* = 8 (SATQOU).

Previous structures of H_2_ *X*_8_-F_20_TPP·solvent have been described for *X* = H (Birnbaum *et al.*, 1995 ▸; Dogutan *et al.*, 2010 ▸), F (Leroy *et al.*, 1999 ▸), Cl (Birnbaum *et al.*, 1995 ▸), and Br (Birnbaum *et al.*, 1995 ▸) (Fig. 4 ▸ *a*). The increasing distortion of macrocycles with increasingly larger halogens can be observed in the plot of skeletal deviations shown in Fig. 4 ▸ *c*.

The macrocycle structures of **1·CHCl_3_** and **1·THF** can be directly compared to the previous structure **1·C_6_H_4_Cl_2_**; these three structures all exhibit approximately the same macrocycle bond distances and angles, shown in Table 3 ▸. The supra­molecular inter­actions of **1·C_6_H_4_Cl_2_** could not be reliably determined given that the solvent was only partially modelled in the reported structure. The face-to-face stacking centroid-to-centroid distance of porphyrin macrocycles in **1·C_6_H_4_Cl_2_** was 6.93 Å, whereas for **1·CHCl_3_**, the separation was 6.83 Å. It is additionally possible that the solvent in the former case was in fact di­chloro­methane, which was present in the crystallization solution and displays a similar Cl⋯Cl separation.

**Figure 5:**
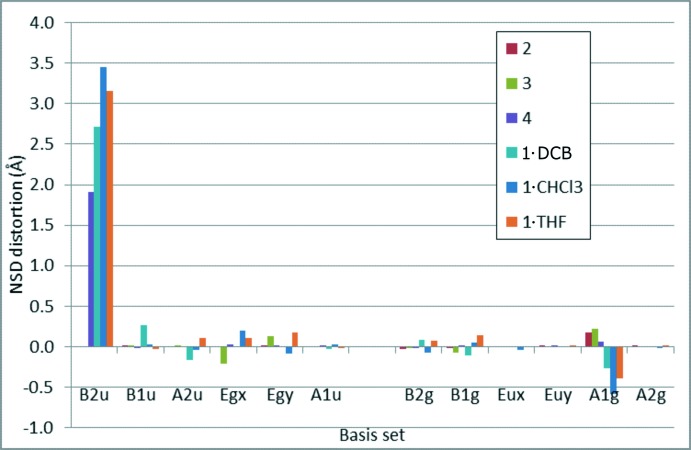
NSD analysis of the *β*-octa-substituted 5,10,15,20-tetra­kis­(penta­fluoro­phen­yl)porphyrin structures H_2_ *X*_8_TPFPP (*X =* H, F, Cl, Br, Br, Br).

The NSD analysis parameters of similar literature structures are summarized in Fig. 5 ▸, as a comparator to the structures in this work. The NSD parameter, in Å, is equal to one quarter of the sum of the displacements of all 24 atoms of the simplified distortion model, which can be attributed to this distortion mode; the error value shown is the sum error (δoop) of the least-squares fit of all six lowest frequency modes. As expected, an increasing saddle-type distortion is found for increasing size of the halogen atom, with little deviation from planarity apparent where *X* = H or F [*sad* = −0.001 (9) Å (H) and 0.000 (0) Å (F)]. Significant saddling distortion was apparent for *X* = Cl [*sad* = 1.91 (2) Å], and greater for *X* = Br [**1·C_6_H_4_Cl_2_** *sad* = 2.72 (5) Å, **1·CHCl_3_** *sad* = 3.45 (7) Å and **1·THF** *sad* = 3.16 (7)], showing the dependence of distortion on steric bulk, which outweighs solvent contributions.

Analogous studies of porphyrins with increasing steric demand have observed increasing distortion for the porphyrin structures (eth­yl)_*x*_-5,10,15,20-tetra­phenyl­porphyrin (*X* = 2, 4, 6, or 8; Fig. 4 ▸ *a*) (Senge & Kalisch, 1997 ▸; Kielmann *et al.*, 2019 ▸); comparatively, this result shows that this manner of distortion is not dependent on inductive contributions from the halogen atoms (Fig. 4 ▸ *a* and 4*d*). The non-fluorinated analogue structure 2,3,7,8,12,13,17,18-octa­bromo-5,10,15,20-tetra­phenyl­porphyrin (**5**, Spyroulias *et al.*, 1997 ▸; Fig. 4 ▸ *a* and Table 3 ▸) shows slightly greater distortion [*sad* = 3.68 (12) Å] and similar bond lengths to the title compounds. These observations would imply that the inductive or steric contribution of the fluoro substituents is negligible in causing increased distortion of the macrocycle.

## Synthesis and crystallization   

This compound was synthesized by a previously reported procedure (Mandon *et al.*, 1992 ▸). Crystallization was performed by slow evaporation of a partially covered homogeneous solution at room temperature; of chloro­form for **1·CHCl_3_** and THF for **1·THF**.

## Refinement   

Crystal data, data collection and structure refinement details are summarized in Table 4 ▸.

Compound **1·CHCl_3_** was refined as an inversion twin, with a Flack parameter of 0.010 (4), indicating a small inversion impurity in the single crystal. The penta­fluoro­phenyl group bound to C5 was modelled as disordered over two equivalent [0.462 (7):0.538 (7)] coplanar positions, displaced by ≃0.3 Å at the centroid, which were constrained to have equal *U^ij^* parameters for atoms sharing sites, similar *U^ij^* parameters for bonded atoms, and idealized ring geometry. The most distant fluorine atoms had to be held to additional isotropic *U^ij^* restraints. The chloro­form solvate was also modelled as disordered over two orientations, sharing approximate carbon and hydrogen positions. This second orientation was related by a partial rotation around the threefold axis and modelled such that these two orientations had a sum occupancy of one mol­ecule. The dominant orientation was refined to 0.882 (7) occupancy, and C—Cl distances in the minor component had to be restrained to idealized bond distances. C atoms were held to equal *U^ij^* restraints and Cl atoms were restrained to similar *U^ij^* parameters.

In compound **1·THF** penta­fluoro­phenyl rings were modelled as disordered over two orientations with dominant orientations of 0.748 (18) and 0.694 (17) occupancy. Porphyrin-to-phenyl distances and carbon atom displacement parameters (*U^ij^*) were restrained. Idealized geometric constraints were imposed on the least occupied phenyl ring C10*A*–C10*F*. The *ipso* phenyl carbon atoms were constrained to have equal *U^ij^* parameters and positions. Pyrrole hydrogen atoms were located in the difference-Fourier map and restrained using idealized bond distances.

## Supplementary Material

Crystal structure: contains datablock(s) 1_CHCl3, 1_THF. DOI: 10.1107/S2056989020000432/tx2016sup1.cif

Structure factors: contains datablock(s) 1_CHCl3. DOI: 10.1107/S2056989020000432/tx20161_CHCl3sup4.hkl

Structure factors: contains datablock(s) 1_THF. DOI: 10.1107/S2056989020000432/tx20161_THFsup5.hkl

CCDC references: 1977525, 1977524

Additional supporting information:  crystallographic information; 3D view; checkCIF report

## Figures and Tables

**Table 1:** Hydrogen-bond geometry (Å, °) for **1·CHCl_3_** [Chem scheme1]

*D*—H⋯*A*	*D*—H	H⋯*A*	*D*⋯*A*	*D*—H⋯*A*
N21—H21⋯N22	0.88	2.34	2.866 (3)	119
N21—H21⋯N24	0.88	2.42	2.928 (3)	117
N23—H23⋯N22	0.88	2.48	2.931 (3)	113
N23—H23⋯N24	0.88	2.46	2.895 (3)	111

**Table 2:** Hydrogen-bond geometry (Å, °) for **1·THF** [Chem scheme1]

*D*—H⋯*A*	*D*—H	H⋯*A*	*D*⋯*A*	*D*—H⋯*A*
N22—H22⋯O25	0.97 (1)	1.92 (2)	2.849 (6)	158 (5)

**Table 3:** Calculated mean distances, angles and structural parameters (Å, °) for compounds 1–6 Measured mean bond distances, angles, mean-plane angles, calculated NSD values, intra­molecular contacts and mean-plane deviations for atom groups.

	Br_8_TPFPP·CHCl_3_	Br_8_TPFPP1·THF	H_8_TPFPP·dioxane	F_8_TPFPP	Cl_8_TPFPP	Br_8_TPFPP·C_6_H_4_Cl_2_	Br_8_TPP·DMF
	**1·CHCl_3_**	**1·THF**	**2**	**3**	**4**	**1·C_6_H_4_Cl_2_**	**6**
	This work	This work	(HALZUP; Dog-	(GODYON; Leroy	(ZALHUP; Birn-	(ZALJEB; Birn-	(RONROB; Spyr-
			utan *et al.*, 2010 ▸)	*et al.*, 1999 ▸)	baum *et al.*, 1995 ▸)	baum *et al.*, 1995 ▸)	oulias *et al.*, 1997 ▸)
Bond lengths							
N—C_a_	1.368 (7)	1.366 (6)	1.358	1.370	1.372	1.367	1.362
C_a_—C_b_	1.440 (20)	1.450 (2)	1.450	1.438	1.448	1.457	1.435
C_a_—C_m_	1.406 (10)	1.408 (4)	1.395	1.399	1.402	1.406	1.415
C_b_—C_b_	1.361 (10)	1.357 (12)	1.345	1.332	1.347	1.349	1.348
							
Bond angles							
N—C_a_—C_m_	123.1 (7)	123.7 (12)	126.1	126.6	125.4	124.5	123.1
N—C_a_—C_b_	110.0 (6)	110.0 (6)	108.8	107.5	107.2	108.1	107.5
C_a_—N—C_a_	109.0 (4)	109.0 (3)	108.1	108.7	109.5	108.7	109.4
C_a_—C_m_—C_a_	123.0 (9)	123.8 (7)	125.8	125.4	125.7	123.4	120.9
C_a_—C_b_—C_b_	107.4 (12)	107.5 (9)	107.1	108.1	108.0	107.4	107.7
C_m_—C_a_—C_b_	129.0 (2)	128.3 (14)	125.0	125.9	127.2	127.1	129.3
							
Pyrrole mean-plane incline angles							
<pyr_N21_	27.8	25.6	0.7	3.1	14.6	25.6	39.1
<pyr_N22_	26.9	29.5	0.4	3.4	15.7	20.4	39.1
<pyr_N23_	36.9	36.5	0.7	3.1	14.9	25.6	39.1
<pyr_N24_	32.1	22.3	0.4	3.4	14.0	20.4	39.1
Mean(<pyr)	30.0	28.5	0.6	3.2	14.8	23.0	39.1
							
Structural parameters							
Δip^*a*^	0.59 (11)	0.42 (10)	0.182 (12)	0.23 (2)	0.07 (4)	0.31 (7)	0.8 (2)
Δoop^*b*^	3.46 (7)	3.17 (7)	0.01 (9)	0.246 (8)	1.91 (2)	2.73 (5)	3.790 (120)
N21⋯N22	2.866 (3)	2.43 (5)	2.927	2.921	2.943	2.923	2.958
N22⋯N23	2.931 (3)	2.32 (5)	2.895	2.942	2.939	2.86	2.958
N23⋯N24	2.895 (3)	2.61 (6)	2.927	2.921	2.942	2.923	2.958
N24⋯N21	2.928 (3)	2.60 (6)	2.895	2.942	2.925	2.86	2.958
Δ24^*c*^	0.543	0.497	0.008	0.046	0.308	0.442	0.616
ΔN^*d*^	0.075	0.090	0.018	0.046	0.088	0.077	0.034
ΔC_m_^*e*^	0.036	0.017	0.009	0.061	0.009	0.091	0.317
ΔC_a_^*f*^	0.418	0.379	0.007	0.029	0.250	0.339	0.409
ΔC_b_^*g*^	1.156	1.060	0.005	0.055	0.626	0.903	1.264

(*a*) Simulated total in-plane distortion; (*b*) simulated total out-of-plane distortion; (*c*) average deviation from the least-squares plane of the 24-macrocycle atoms; (*d*) simulated displacement of the four inter­nal nitro­gen atoms from the 24-atom mean plane; (*e*) average deviation of the *meso*-carbon atoms from the 24-atom mean plane; (*f*) Average deviation of the α-carbon atoms from the 24-atom mean plane; (*g*) Average deviation of the β-carbon atoms from the 24-atom mean plane.

**Table 4:** Experimental details

	**1·CHCl_3_**	**1·THF**
Crystal data		
Chemical formula	C_44_H_2_Br_8_F_20_N_4_·CHCl_3_	C_44_H_2_Br_8_F_20_N_4_·C_4_H_8_O
*M*_r_	1725.14	1677.88
Crystal system, space group	Monoclinic, *P*2_1_	Monoclinic, *P*2_1_/*n*
Temperature (K)	100	100
*a*, *b*, *c* (Å)	15.5162 (7), 6.8288 (3), 24.5631 (12)	21.524 (9), 9.545 (4), 26.020 (11)
β (°)	104.683 (1)	107.426 (7)
*V* (Å^3^)	2517.6 (2)	5101 (4)
*Z*	2	4
Radiation type	Mo *K*α	Mo *K*α
μ (mm^−1^)	6.65	6.41
Crystal size (mm)	0.31 × 0.30 × 0.11	0.42 × 0.11 × 0.09

Data collection		
Diffractometer	Bruker APEXII CCD	Bruker APEXII CCD
Absorption correction	Multi-scan (*SADABS*; Bruker, 2016 ▸)	Multi-scan (*SADABS*; Bruker, 2016 ▸)
*T*_min_, *T*_max_	0.458, 0.746	0.455, 0.746
No. of measured, independent and observed [*I* > 2σ(*I*)] reflections	107677, 18291, 17164	148293, 12689, 10079
*R*_int_	0.028	0.082
(sin θ/λ)_max_ (Å^−1^)	0.758	0.668

Refinement		
*R*[*F* ^2^ > 2σ(*F* ^2^)], *wR*(*F* ^2^), *S*	0.020, 0.043, 1.03	0.054, 0.095, 1.21
No. of reflections	18291	12689
No. of parameters	763	908
No. of restraints	244	718
H-atom treatment	H-atom parameters constrained	H atoms treated by a mixture of independent and constrained refinement
		
Δρ_max_, Δρ_min_ (e Å^−3^)	0.52, −0.46	1.63, −0.98
Absolute structure	Refined as an inversion twin.	–
Absolute structure parameter	0.010 (4)	–

Computer programs: *APEX3* (Bruker, 2017 ▸), *SAINT* (Bruker, 2015 ▸), *SHELXT* (Sheldrick, 2015*a* ▸), *SHELXL* (Sheldrick, 2015*b* ▸), *Shelxle* (Hübschle *et al.*, 2011 ▸) and *OLEX2* (Dolomanov *et al.*, 2009 ▸).

## supplementary crystallographic information

### 2,3,7,8,12,13,17,18-Octabromo-5,10,15,20-tetrakis(pentafluorophenyl)porphyrin chloroform monosolvate (1_CHCl3) Crystal data

**Table:**

C_44_H_2_Br_8_F_20_N_4_·CHCl_3_	*F*(000) = 1624
*M**_r_* = 1725.14	*D*_x_ = 2.276 Mg m^−^^3^
Monoclinic, *P*2_1_	Mo *K*α radiation, λ = 0.71073 Å
*a* = 15.5162 (7) Å	Cell parameters from 9974 reflections
*b* = 6.8288 (3) Å	θ = 2.6–32.5°
*c* = 24.5631 (12) Å	µ = 6.65 mm^−^^1^
β = 104.683 (1)°	*T* = 100 K
*V* = 2517.6 (2) Å^3^	Block, blue
*Z* = 2	0.31 × 0.30 × 0.11 mm

### 2,3,7,8,12,13,17,18-Octabromo-5,10,15,20-tetrakis(pentafluorophenyl)porphyrin chloroform monosolvate (1_CHCl3) Data collection

**Table:**

Bruker APEXII CCD diffractometer	18291 independent reflections
Radiation source: microfocus sealed X-ray tube, graphite-monochromated	17164 reflections with *I* > 2σ(*I*)
Mirror optics monochromator	*R*_int_ = 0.028
Detector resolution: 7.9 pixels mm^-1^	θ_max_ = 32.6°, θ_min_ = 1.4°
φ and ω scans	*h* = −23→23
Absorption correction: multi-scan (SADABS; Bruker, 2016)	*k* = −10→10
*T*_min_ = 0.458, *T*_max_ = 0.746	*l* = −37→36
107677 measured reflections	

### 2,3,7,8,12,13,17,18-Octabromo-5,10,15,20-tetrakis(pentafluorophenyl)porphyrin chloroform monosolvate (1_CHCl3) Refinement

**Table:**

Refinement on *F*^2^	Hydrogen site location: inferred from neighbouring sites
Least-squares matrix: full	H-atom parameters constrained
*R*[*F*^2^ > 2σ(*F*^2^)] = 0.020	*w* = 1/[σ^2^(*F*_o_^2^) + (0.0025*P*)^2^ + 1.005*P*] where *P* = (*F*_o_^2^ + 2*F*_c_^2^)/3
*wR*(*F*^2^) = 0.043	(Δ/σ)_max_ = 0.002
*S* = 1.03	Δρ_max_ = 0.52 e Å^−^^3^
18291 reflections	Δρ_min_ = −0.46 e Å^−^^3^
763 parameters	Absolute structure: Refined as an inversion twin.
244 restraints	Absolute structure parameter: 0.010 (4)
Primary atom site location: dual	

### 2,3,7,8,12,13,17,18-Octabromo-5,10,15,20-tetrakis(pentafluorophenyl)porphyrin chloroform monosolvate (1_CHCl3) Special details

**Table:**

Geometry. All esds (except the esd in the dihedral angle between two l.s. planes) are estimated using the full covariance matrix. The cell esds are taken into account individually in the estimation of esds in distances, angles and torsion angles; correlations between esds in cell parameters are only used when they are defined by crystal symmetry. An approximate (isotropic) treatment of cell esds is used for estimating esds involving l.s. planes.

### 2,3,7,8,12,13,17,18-Octabromo-5,10,15,20-tetrakis(pentafluorophenyl)porphyrin chloroform monosolvate (1_CHCl3) Fractional atomic coordinates and isotropic or equivalent isotropic displacement parameters (Å^2^)

**Table:**

	*x*	*y*	*z*	*U*_iso_*/*U*_eq_	Occ. (<1)
C1	0.79032 (16)	0.5406 (4)	0.87273 (10)	0.0134 (4)	
Br1	0.87104 (2)	0.43518 (5)	0.99180 (2)	0.02349 (6)	
C2	0.86821 (16)	0.5046 (4)	0.91809 (10)	0.0152 (4)	
Br2	1.05536 (2)	0.43897 (5)	0.93835 (2)	0.02288 (6)	
C3	0.94138 (16)	0.5053 (4)	0.89661 (10)	0.0153 (4)	
Br3	1.06413 (2)	0.81653 (4)	0.70329 (2)	0.02160 (6)	
C4	0.91257 (15)	0.5427 (4)	0.83707 (10)	0.0132 (4)	
Br4	0.92250 (2)	0.68017 (4)	0.57534 (2)	0.01783 (5)	
C5	0.96138 (16)	0.5753 (4)	0.79756 (10)	0.0145 (4)	
Br5	0.67971 (2)	−0.08433 (4)	0.56768 (2)	0.02307 (5)	
C6	0.92364 (15)	0.5776 (4)	0.73877 (10)	0.0134 (4)	
F6	0.94084 (10)	0.1746 (3)	0.61796 (6)	0.0201 (3)	
Br6	0.49352 (2)	−0.08753 (4)	0.62177 (2)	0.02119 (5)	
C7	0.96548 (16)	0.6523 (4)	0.69563 (10)	0.0154 (4)	
F7	0.98329 (10)	0.0661 (3)	0.52270 (7)	0.0227 (3)	
Br7	0.37497 (2)	0.69017 (4)	0.73639 (2)	0.01604 (5)	
C8	0.91110 (16)	0.5994 (4)	0.64558 (10)	0.0139 (4)	
F8	0.86376 (11)	0.1210 (3)	0.42128 (7)	0.0246 (4)	
Br8	0.51364 (2)	0.86107 (4)	0.86350 (2)	0.01969 (5)	
C9	0.83861 (15)	0.4866 (3)	0.65798 (10)	0.0133 (4)	
F9	0.70704 (11)	0.3005 (3)	0.41561 (6)	0.0247 (3)	
C10	0.77912 (15)	0.3636 (4)	0.62036 (10)	0.0139 (4)	
F10	0.66599 (10)	0.4240 (3)	0.51083 (6)	0.0229 (3)	
C11	0.70159 (15)	0.2861 (4)	0.63184 (10)	0.0137 (4)	
F11	0.41703 (10)	0.4175 (3)	0.58292 (6)	0.0224 (3)	
C12	0.64841 (16)	0.1194 (4)	0.60940 (10)	0.0150 (4)	
F12	0.24742 (11)	0.2960 (3)	0.54385 (7)	0.0268 (4)	
C13	0.57470 (16)	0.1181 (4)	0.63059 (10)	0.0149 (4)	
F13	0.16081 (11)	0.1268 (3)	0.61447 (9)	0.0321 (4)	
C14	0.57932 (15)	0.2834 (4)	0.66693 (10)	0.0130 (4)	
F14	0.24506 (11)	0.0721 (3)	0.72457 (8)	0.0270 (4)	
C15	0.51835 (15)	0.3577 (3)	0.69576 (10)	0.0127 (4)	
F15	0.41634 (10)	0.1838 (2)	0.76383 (6)	0.0196 (3)	
C16	0.54601 (15)	0.4764 (3)	0.74374 (10)	0.0126 (4)	
F16	0.59460 (13)	0.3120 (3)	0.91627 (7)	0.0328 (4)	
C17	0.49068 (15)	0.6028 (3)	0.76885 (10)	0.0138 (4)	
F17	0.54619 (14)	0.3700 (4)	1.01268 (8)	0.0471 (6)	
C18	0.54346 (15)	0.6649 (4)	0.81857 (10)	0.0141 (4)	
F18	0.60364 (13)	0.6916 (4)	1.07618 (7)	0.0433 (5)	
C19	0.63021 (15)	0.5740 (4)	0.82428 (10)	0.0133 (4)	
F19	0.71156 (14)	0.9551 (4)	1.04248 (8)	0.0435 (5)	
C20	0.70187 (16)	0.5724 (4)	0.87352 (10)	0.0140 (4)	
F20	0.76215 (12)	0.8962 (3)	0.94747 (8)	0.0315 (4)	
N21	0.82117 (13)	0.5520 (3)	0.82522 (8)	0.0122 (3)	
H21	0.786401	0.563972	0.790997	0.015*	
N22	0.84463 (13)	0.4908 (3)	0.71443 (8)	0.0129 (4)	
N23	0.65875 (13)	0.3736 (3)	0.66794 (8)	0.0136 (4)	
H23	0.679701	0.475296	0.689186	0.016*	
Cl1S	0.8289 (3)	1.0578 (6)	0.84825 (15)	0.0405 (6)	0.882 (7)
F5	1.0834 (12)	0.304 (4)	0.7761 (9)	0.0289 (15)	0.462 (7)
F4	1.2643 (6)	0.3383 (11)	0.8118 (4)	0.0363 (11)	0.462 (7)
F3	1.3335 (5)	0.6633 (11)	0.8705 (3)	0.0379 (10)	0.462 (7)
F2	1.2238 (5)	0.9445 (11)	0.8947 (3)	0.0382 (11)	0.462 (7)
F1	1.0460 (9)	0.900 (3)	0.8631 (7)	0.0303 (14)	0.462 (7)
C1S	0.7822 (3)	1.0817 (7)	0.7756 (2)	0.0287 (9)	0.882 (7)
H	0.762829	1.220837	0.767873	0.034*	0.882 (7)
Cl2S	0.68818 (11)	0.9313 (2)	0.75235 (8)	0.0489 (5)	0.882 (7)
Cl3S	0.86152 (16)	1.0294 (2)	0.73764 (8)	0.0461 (4)	0.882 (7)
N24	0.63172 (13)	0.4721 (3)	0.77706 (8)	0.0132 (4)	
C51	1.0608 (6)	0.603 (3)	0.8183 (10)	0.0210 (7)	0.462 (7)
C52	1.0984 (7)	0.767 (2)	0.8488 (8)	0.0255 (12)	0.462 (7)
C53	1.1905 (7)	0.7875 (14)	0.8658 (6)	0.0287 (14)	0.462 (7)
C54	1.2450 (6)	0.6439 (12)	0.8522 (5)	0.0292 (14)	0.462 (7)
C55	1.2074 (9)	0.4800 (15)	0.8217 (6)	0.0280 (13)	0.462 (7)
C56	1.1154 (9)	0.460 (2)	0.8047 (8)	0.0241 (9)	0.462 (7)
C2S	0.770 (2)	1.107 (6)	0.7747 (14)	0.0287 (9)	0.118 (7)
HA	0.763429	1.250725	0.766630	0.034*	0.118 (7)
C51B	1.0594 (5)	0.616 (2)	0.8184 (8)	0.0210 (7)	0.538 (7)
C52B	1.0853 (6)	0.793 (2)	0.8456 (7)	0.0255 (12)	0.538 (7)
C53B	1.1752 (6)	0.8393 (12)	0.8646 (5)	0.0287 (14)	0.538 (7)
C54B	1.2393 (5)	0.7081 (11)	0.8564 (4)	0.0292 (14)	0.538 (7)
C55B	1.2134 (7)	0.5311 (12)	0.8292 (5)	0.0280 (13)	0.538 (7)
C56B	1.1235 (8)	0.4852 (18)	0.8102 (7)	0.0241 (9)	0.538 (7)
F1B	1.0276 (8)	0.927 (2)	0.8547 (6)	0.0303 (14)	0.538 (7)
F2B	1.2028 (4)	1.0127 (9)	0.8910 (3)	0.0382 (11)	0.538 (7)
F3B	1.3255 (4)	0.7480 (10)	0.8757 (3)	0.0379 (10)	0.538 (7)
F4B	1.2721 (5)	0.4032 (10)	0.8213 (3)	0.0363 (11)	0.538 (7)
F5B	1.0987 (10)	0.315 (4)	0.7843 (8)	0.0289 (15)	0.538 (7)
C101	0.80163 (15)	0.3017 (4)	0.56770 (10)	0.0137 (4)	
C102	0.88211 (16)	0.2099 (4)	0.56870 (10)	0.0148 (4)	
C103	0.90484 (16)	0.1527 (4)	0.52009 (11)	0.0158 (4)	
C104	0.84515 (17)	0.1824 (4)	0.46866 (10)	0.0180 (4)	
C105	0.76466 (16)	0.2730 (4)	0.46601 (10)	0.0171 (5)	
C106	0.74383 (15)	0.3321 (4)	0.51480 (10)	0.0159 (4)	
C151	0.42329 (15)	0.3018 (4)	0.67476 (10)	0.0127 (4)	
C152	0.37698 (16)	0.3310 (4)	0.61870 (10)	0.0156 (4)	
C153	0.28927 (17)	0.2728 (4)	0.59851 (11)	0.0188 (5)	
C154	0.24496 (15)	0.1857 (4)	0.63424 (12)	0.0206 (5)	
C155	0.28786 (16)	0.1582 (4)	0.69014 (12)	0.0189 (5)	
C156	0.37625 (16)	0.2150 (4)	0.70987 (10)	0.0149 (4)	
C201	0.67914 (16)	0.6031 (4)	0.92807 (10)	0.0167 (5)	
C202	0.62418 (17)	0.4721 (4)	0.94653 (11)	0.0214 (5)	
C203	0.59867 (19)	0.5003 (5)	0.99606 (12)	0.0291 (7)	
C204	0.62785 (19)	0.6622 (6)	1.02837 (11)	0.0306 (7)	
C205	0.68316 (19)	0.7956 (5)	1.01145 (12)	0.0276 (6)	
C206	0.70860 (18)	0.7645 (4)	0.96233 (11)	0.0212 (5)	
Cl4S	0.8187 (18)	0.975 (3)	0.7296 (6)	0.069 (4)	0.118 (7)
Cl5S	0.6705 (9)	0.989 (2)	0.7786 (10)	0.068 (5)	0.118 (7)
Cl6S	0.839 (2)	1.047 (5)	0.8409 (12)	0.049 (6)	0.118 (7)

### 2,3,7,8,12,13,17,18-Octabromo-5,10,15,20-tetrakis(pentafluorophenyl)porphyrin chloroform monosolvate (1_CHCl3) Atomic displacement parameters (Å^2^)

**Table:**

	*U*^11^	*U*^22^	*U*^33^	*U*^12^	*U*^13^	*U*^23^
C1	0.0128 (10)	0.0167 (10)	0.0107 (10)	−0.0011 (8)	0.0027 (8)	0.0002 (8)
Br1	0.02061 (12)	0.03766 (15)	0.01195 (10)	0.00155 (11)	0.00370 (9)	0.00789 (11)
C2	0.0145 (11)	0.0201 (11)	0.0109 (10)	−0.0009 (8)	0.0028 (8)	0.0020 (8)
Br2	0.01293 (10)	0.03700 (15)	0.01655 (11)	0.00356 (11)	−0.00026 (8)	0.00561 (11)
C3	0.0113 (10)	0.0210 (11)	0.0123 (10)	−0.0005 (8)	0.0005 (8)	0.0023 (8)
Br3	0.01564 (11)	0.03080 (14)	0.01819 (12)	−0.01240 (10)	0.00399 (9)	0.00174 (10)
C4	0.0095 (9)	0.0170 (10)	0.0118 (10)	−0.0019 (8)	0.0001 (8)	0.0004 (8)
Br4	0.01723 (11)	0.02391 (11)	0.01397 (10)	−0.00445 (10)	0.00694 (8)	0.00244 (9)
C5	0.0118 (10)	0.0193 (11)	0.0127 (10)	−0.0041 (8)	0.0037 (8)	−0.0012 (8)
Br5	0.02230 (12)	0.02245 (12)	0.02858 (13)	−0.00561 (10)	0.01408 (10)	−0.01180 (10)
C6	0.0099 (9)	0.0176 (10)	0.0126 (10)	−0.0019 (8)	0.0023 (8)	0.0002 (8)
F6	0.0148 (6)	0.0309 (8)	0.0135 (7)	0.0038 (7)	0.0017 (5)	0.0026 (6)
Br6	0.01599 (11)	0.01999 (12)	0.02962 (13)	−0.00840 (10)	0.00952 (9)	−0.00895 (10)
C7	0.0120 (10)	0.0205 (12)	0.0142 (10)	−0.0053 (8)	0.0041 (8)	0.0001 (8)
F7	0.0154 (7)	0.0339 (9)	0.0219 (8)	0.0074 (7)	0.0103 (6)	0.0043 (7)
Br7	0.01161 (10)	0.01818 (10)	0.01770 (11)	0.00211 (9)	0.00253 (8)	−0.00019 (9)
C8	0.0120 (10)	0.0191 (11)	0.0116 (10)	−0.0025 (8)	0.0047 (8)	0.0023 (8)
F8	0.0231 (8)	0.0395 (10)	0.0144 (7)	0.0028 (7)	0.0105 (6)	−0.0013 (6)
Br8	0.01666 (11)	0.02534 (12)	0.01741 (11)	0.00252 (9)	0.00493 (9)	−0.00738 (9)
C9	0.0100 (10)	0.0175 (10)	0.0128 (10)	−0.0026 (8)	0.0037 (8)	−0.0001 (8)
F9	0.0191 (7)	0.0417 (10)	0.0109 (7)	0.0042 (7)	−0.0004 (6)	0.0005 (7)
C10	0.0106 (9)	0.0192 (10)	0.0121 (10)	−0.0008 (8)	0.0035 (8)	0.0003 (8)
F10	0.0136 (6)	0.0366 (9)	0.0176 (7)	0.0075 (7)	0.0026 (5)	0.0001 (7)
C11	0.0114 (10)	0.0179 (11)	0.0119 (10)	−0.0016 (8)	0.0033 (8)	−0.0017 (8)
F11	0.0190 (7)	0.0339 (9)	0.0147 (7)	−0.0046 (7)	0.0046 (5)	0.0036 (7)
C12	0.0127 (10)	0.0191 (10)	0.0141 (10)	−0.0018 (8)	0.0049 (8)	−0.0044 (8)
F12	0.0194 (8)	0.0364 (9)	0.0186 (8)	−0.0014 (7)	−0.0059 (6)	0.0008 (7)
C13	0.0113 (10)	0.0174 (10)	0.0163 (11)	−0.0050 (8)	0.0037 (8)	−0.0035 (8)
F13	0.0092 (7)	0.0391 (10)	0.0436 (11)	−0.0090 (7)	−0.0015 (7)	0.0024 (8)
C14	0.0106 (9)	0.0178 (11)	0.0108 (10)	−0.0036 (8)	0.0029 (8)	−0.0016 (8)
F14	0.0167 (7)	0.0310 (9)	0.0364 (10)	−0.0066 (7)	0.0128 (7)	0.0085 (7)
C15	0.0100 (9)	0.0160 (10)	0.0117 (10)	−0.0029 (8)	0.0024 (7)	0.0003 (8)
F15	0.0187 (7)	0.0241 (7)	0.0160 (7)	−0.0034 (7)	0.0044 (5)	0.0046 (6)
C16	0.0094 (9)	0.0155 (10)	0.0132 (10)	−0.0019 (8)	0.0035 (8)	0.0008 (8)
F16	0.0387 (10)	0.0377 (10)	0.0246 (9)	−0.0199 (8)	0.0130 (8)	−0.0029 (8)
C17	0.0121 (10)	0.0155 (10)	0.0142 (10)	−0.0010 (8)	0.0043 (8)	0.0008 (8)
F17	0.0390 (11)	0.0824 (18)	0.0253 (9)	−0.0214 (12)	0.0179 (8)	0.0062 (10)
C18	0.0129 (10)	0.0165 (11)	0.0142 (10)	−0.0020 (8)	0.0061 (8)	−0.0021 (8)
F18	0.0316 (10)	0.0870 (17)	0.0141 (8)	0.0069 (11)	0.0110 (7)	−0.0063 (10)
C19	0.0115 (10)	0.0166 (10)	0.0119 (10)	−0.0015 (8)	0.0031 (8)	−0.0009 (8)
F19	0.0423 (11)	0.0587 (14)	0.0298 (10)	−0.0051 (10)	0.0096 (8)	−0.0288 (10)
C20	0.0130 (10)	0.0177 (10)	0.0114 (10)	−0.0029 (8)	0.0030 (8)	−0.0009 (8)
F20	0.0314 (9)	0.0332 (10)	0.0319 (9)	−0.0137 (8)	0.0119 (7)	−0.0134 (8)
N21	0.0085 (8)	0.0167 (9)	0.0105 (9)	−0.0010 (7)	0.0008 (7)	0.0010 (7)
N22	0.0103 (8)	0.0171 (9)	0.0109 (9)	−0.0028 (7)	0.0022 (7)	−0.0005 (7)
N23	0.0102 (8)	0.0179 (9)	0.0137 (9)	−0.0050 (7)	0.0048 (7)	−0.0047 (7)
Cl1S	0.0756 (12)	0.0204 (8)	0.0225 (8)	0.0033 (7)	0.0072 (7)	0.0020 (7)
F5	0.011 (4)	0.043 (2)	0.030 (5)	0.008 (4)	0.001 (3)	0.002 (3)
F4	0.0166 (13)	0.053 (3)	0.041 (2)	0.007 (2)	0.0093 (14)	0.006 (2)
F3	0.0136 (12)	0.066 (3)	0.0308 (14)	−0.014 (2)	−0.0003 (9)	0.008 (2)
F2	0.030 (2)	0.051 (3)	0.0291 (13)	−0.0231 (19)	0.0003 (16)	−0.007 (2)
F1	0.026 (4)	0.038 (4)	0.024 (4)	−0.012 (3)	0.001 (3)	−0.009 (2)
C1S	0.044 (2)	0.0133 (18)	0.0273 (15)	0.0029 (14)	0.0058 (15)	0.0024 (12)
Cl2S	0.0507 (7)	0.0301 (6)	0.0534 (9)	−0.0098 (5)	−0.0100 (6)	0.0206 (6)
Cl3S	0.0720 (11)	0.0283 (6)	0.0474 (8)	0.0150 (6)	0.0326 (8)	0.0138 (5)
N24	0.0100 (8)	0.0178 (9)	0.0117 (9)	−0.0008 (7)	0.0027 (7)	−0.0017 (7)
C51	0.0106 (10)	0.038 (2)	0.0130 (11)	−0.0062 (11)	0.0011 (9)	0.0038 (14)
C52	0.014 (2)	0.047 (3)	0.0141 (19)	−0.012 (2)	0.000 (2)	0.001 (2)
C53	0.019 (3)	0.051 (4)	0.0150 (13)	−0.016 (3)	0.0008 (19)	0.001 (3)
C54	0.0128 (14)	0.055 (4)	0.0175 (18)	−0.012 (3)	−0.0001 (13)	0.007 (3)
C55	0.0096 (16)	0.055 (4)	0.019 (3)	−0.004 (3)	0.0040 (17)	0.008 (3)
C56	0.0091 (19)	0.047 (3)	0.015 (3)	−0.0042 (16)	−0.0001 (16)	0.0050 (19)
C2S	0.044 (2)	0.0133 (18)	0.0273 (15)	0.0029 (14)	0.0058 (15)	0.0024 (12)
C51B	0.0106 (10)	0.038 (2)	0.0130 (11)	−0.0062 (11)	0.0011 (9)	0.0038 (14)
C52B	0.014 (2)	0.047 (3)	0.0141 (19)	−0.012 (2)	0.000 (2)	0.001 (2)
C53B	0.019 (3)	0.051 (4)	0.0150 (13)	−0.016 (3)	0.0008 (19)	0.001 (3)
C54B	0.0128 (14)	0.055 (4)	0.0175 (18)	−0.012 (3)	−0.0001 (13)	0.007 (3)
C55B	0.0096 (16)	0.055 (4)	0.019 (3)	−0.004 (3)	0.0040 (17)	0.008 (3)
C56B	0.0091 (19)	0.047 (3)	0.015 (3)	−0.0042 (16)	−0.0001 (16)	0.0050 (19)
F1B	0.026 (4)	0.038 (4)	0.024 (4)	−0.012 (3)	0.001 (3)	−0.009 (2)
F2B	0.030 (2)	0.051 (3)	0.0291 (13)	−0.0231 (19)	0.0003 (16)	−0.007 (2)
F3B	0.0136 (12)	0.066 (3)	0.0308 (14)	−0.014 (2)	−0.0003 (9)	0.008 (2)
F4B	0.0166 (13)	0.053 (3)	0.041 (2)	0.007 (2)	0.0093 (14)	0.006 (2)
F5B	0.011 (4)	0.043 (2)	0.030 (5)	0.008 (4)	0.001 (3)	0.002 (3)
C101	0.0109 (9)	0.0190 (10)	0.0118 (10)	−0.0015 (8)	0.0039 (8)	−0.0007 (8)
C102	0.0121 (10)	0.0187 (11)	0.0137 (10)	−0.0012 (8)	0.0036 (8)	0.0011 (8)
C103	0.0127 (10)	0.0203 (12)	0.0165 (11)	0.0011 (8)	0.0076 (8)	0.0024 (8)
C104	0.0187 (11)	0.0232 (11)	0.0139 (10)	−0.0010 (10)	0.0078 (8)	−0.0004 (10)
C105	0.0143 (11)	0.0254 (12)	0.0108 (10)	−0.0016 (9)	0.0017 (8)	0.0001 (9)
C106	0.0113 (10)	0.0204 (11)	0.0166 (11)	−0.0007 (8)	0.0047 (8)	−0.0004 (9)
C151	0.0095 (9)	0.0155 (10)	0.0134 (10)	−0.0018 (8)	0.0034 (8)	−0.0021 (8)
C152	0.0137 (10)	0.0190 (11)	0.0141 (10)	−0.0026 (9)	0.0036 (8)	−0.0008 (8)
C153	0.0146 (11)	0.0212 (12)	0.0178 (12)	0.0003 (9)	−0.0011 (9)	−0.0020 (9)
C154	0.0071 (9)	0.0209 (11)	0.0314 (13)	−0.0036 (9)	0.0003 (9)	−0.0004 (11)
C155	0.0119 (10)	0.0183 (12)	0.0284 (13)	−0.0023 (9)	0.0087 (9)	0.0030 (10)
C156	0.0129 (10)	0.0165 (11)	0.0161 (11)	−0.0003 (8)	0.0053 (8)	0.0005 (8)
C201	0.0119 (10)	0.0268 (12)	0.0109 (10)	−0.0014 (9)	0.0022 (8)	−0.0014 (9)
C202	0.0163 (11)	0.0328 (14)	0.0151 (11)	−0.0054 (10)	0.0040 (9)	0.0002 (10)
C203	0.0191 (13)	0.054 (2)	0.0155 (12)	−0.0046 (12)	0.0070 (10)	0.0053 (12)
C204	0.0180 (12)	0.063 (2)	0.0108 (11)	0.0059 (14)	0.0043 (9)	−0.0027 (13)
C205	0.0209 (13)	0.0445 (17)	0.0154 (12)	0.0019 (12)	0.0010 (10)	−0.0117 (12)
C206	0.0163 (11)	0.0303 (13)	0.0172 (12)	−0.0024 (10)	0.0045 (9)	−0.0051 (10)
Cl4S	0.123 (11)	0.048 (7)	0.037 (5)	0.045 (7)	0.024 (7)	0.015 (5)
Cl5S	0.052 (6)	0.044 (6)	0.097 (10)	−0.017 (5)	−0.003 (6)	0.036 (6)
Cl6S	0.075 (10)	0.025 (5)	0.032 (8)	−0.003 (7)	−0.015 (8)	−0.010 (6)

### 2,3,7,8,12,13,17,18-Octabromo-5,10,15,20-tetrakis(pentafluorophenyl)porphyrin chloroform monosolvate (1_CHCl3) Geometric parameters (Å, º)

**Table:**

C1—C2	1.442 (3)	F19—C205	1.339 (4)
C1—C20	1.394 (3)	C20—C201	1.484 (3)
C1—N21	1.371 (3)	F20—C206	1.336 (3)
Br1—C2	1.861 (2)	N21—H21	0.8800
C2—C3	1.368 (3)	N23—H23	0.8800
Br2—C3	1.862 (2)	Cl1S—C1S	1.755 (5)
C3—C4	1.439 (3)	F5—C56	1.30 (3)
Br3—C7	1.868 (2)	F4—C55	1.372 (13)
C4—C5	1.392 (3)	F3—C54	1.338 (11)
C4—N21	1.375 (3)	F2—C53	1.317 (11)
Br4—C8	1.862 (2)	F1—C52	1.32 (2)
C5—C6	1.414 (3)	C1S—H	1.0000
C5—C51	1.510 (9)	C1S—Cl2S	1.757 (5)
C5—C51B	1.504 (8)	C1S—Cl3S	1.760 (5)
Br5—C12	1.864 (2)	C51—C52	1.3900
C6—C7	1.467 (3)	C51—C56	1.3900
C6—N22	1.357 (3)	C52—C53	1.3900
F6—C102	1.339 (3)	C53—C54	1.3900
Br6—C13	1.862 (2)	C54—C55	1.3900
C7—C8	1.352 (3)	C55—C56	1.3900
F7—C103	1.340 (3)	C2S—HA	1.0000
Br7—C17	1.868 (2)	C2S—Cl4S	1.75 (3)
C8—C9	1.458 (3)	C2S—Cl5S	1.76 (3)
F8—C104	1.336 (3)	C2S—Cl6S	1.76 (3)
Br8—C18	1.867 (2)	C51B—C52B	1.3900
C9—C10	1.407 (3)	C51B—C56B	1.3900
C9—N22	1.366 (3)	C52B—C53B	1.3900
F9—C105	1.344 (3)	C52B—F1B	1.335 (16)
C10—C11	1.407 (3)	C53B—C54B	1.3900
C10—C101	1.484 (3)	C53B—F2B	1.366 (10)
F10—C106	1.343 (3)	C54B—C55B	1.3900
C11—C12	1.432 (3)	C54B—F3B	1.330 (9)
C11—N23	1.372 (3)	C55B—C56B	1.3900
F11—C152	1.336 (3)	C55B—F4B	1.312 (11)
C12—C13	1.371 (3)	C56B—F5B	1.34 (2)
F12—C153	1.344 (3)	C101—C102	1.392 (3)
C13—C14	1.430 (3)	C101—C106	1.395 (3)
F13—C154	1.335 (3)	C102—C103	1.384 (3)
C14—C15	1.412 (3)	C103—C104	1.379 (3)
C14—N23	1.372 (3)	C104—C105	1.380 (4)
F14—C155	1.337 (3)	C105—C106	1.378 (3)
C15—C16	1.405 (3)	C151—C152	1.397 (3)
C15—C151	1.484 (3)	C151—C156	1.395 (3)
F15—C156	1.331 (3)	C152—C153	1.383 (3)
C16—C17	1.460 (3)	C153—C154	1.380 (4)
C16—N24	1.374 (3)	C154—C155	1.378 (4)
F16—C202	1.336 (3)	C155—C156	1.389 (3)
C17—C18	1.354 (3)	C201—C202	1.389 (4)
F17—C203	1.338 (4)	C201—C206	1.391 (4)
C18—C19	1.456 (3)	C202—C203	1.385 (4)
F18—C204	1.336 (3)	C203—C204	1.369 (5)
C19—C20	1.420 (3)	C204—C205	1.385 (5)
C19—N24	1.358 (3)	C205—C206	1.378 (4)
			
C20—C1—C2	130.6 (2)	F3—C54—C55	121.0 (6)
N21—C1—C2	105.2 (2)	C55—C54—C53	120.0
N21—C1—C20	124.1 (2)	F4—C55—C54	117.6 (8)
C1—C2—Br1	127.12 (18)	F4—C55—C56	122.4 (8)
C3—C2—C1	108.4 (2)	C54—C55—C56	120.0
C3—C2—Br1	123.99 (19)	F5—C56—C51	122.3 (14)
C2—C3—Br2	123.52 (19)	F5—C56—C55	117.7 (14)
C2—C3—C4	108.6 (2)	C55—C56—C51	120.0
C4—C3—Br2	127.57 (19)	Cl4S—C2S—HA	114.7
C5—C4—C3	130.8 (2)	Cl4S—C2S—Cl5S	109 (2)
N21—C4—C3	105.2 (2)	Cl4S—C2S—Cl6S	102 (2)
N21—C4—C5	124.0 (2)	Cl5S—C2S—HA	114.7
C4—C5—C6	123.8 (2)	Cl6S—C2S—HA	114.7
C4—C5—C51	118.4 (10)	Cl6S—C2S—Cl5S	100 (2)
C4—C5—C51B	118.3 (8)	C52B—C51B—C5	118.1 (9)
C6—C5—C51	117.8 (10)	C52B—C51B—C56B	120.0
C6—C5—C51B	117.9 (8)	C56B—C51B—C5	121.9 (9)
C5—C6—C7	126.6 (2)	C53B—C52B—C51B	120.0
N22—C6—C5	122.6 (2)	F1B—C52B—C51B	123.4 (10)
N22—C6—C7	110.5 (2)	F1B—C52B—C53B	116.6 (10)
C6—C7—Br3	129.58 (18)	C52B—C53B—C54B	120.0
C8—C7—Br3	123.96 (18)	F2B—C53B—C52B	121.4 (6)
C8—C7—C6	106.0 (2)	F2B—C53B—C54B	118.6 (6)
C7—C8—Br4	125.40 (18)	C53B—C54B—C55B	120.0
C7—C8—C9	106.7 (2)	F3B—C54B—C53B	120.5 (5)
C9—C8—Br4	127.57 (18)	F3B—C54B—C55B	119.5 (5)
C10—C9—C8	126.2 (2)	C54B—C55B—C56B	120.0
N22—C9—C8	110.1 (2)	F4B—C55B—C54B	121.6 (6)
N22—C9—C10	122.9 (2)	F4B—C55B—C56B	118.4 (7)
C9—C10—C101	118.8 (2)	C55B—C56B—C51B	120.0
C11—C10—C9	123.0 (2)	F5B—C56B—C51B	120.0 (11)
C11—C10—C101	118.0 (2)	F5B—C56B—C55B	120.0 (11)
C10—C11—C12	131.3 (2)	C102—C101—C10	121.4 (2)
N23—C11—C10	123.0 (2)	C102—C101—C106	116.5 (2)
N23—C11—C12	105.6 (2)	C106—C101—C10	122.1 (2)
C11—C12—Br5	126.88 (18)	F6—C102—C101	119.9 (2)
C13—C12—Br5	124.21 (19)	F6—C102—C103	117.7 (2)
C13—C12—C11	108.3 (2)	C103—C102—C101	122.4 (2)
C12—C13—Br6	124.44 (19)	F7—C103—C102	120.7 (2)
C12—C13—C14	108.5 (2)	F7—C103—C104	120.0 (2)
C14—C13—Br6	126.40 (18)	C104—C103—C102	119.3 (2)
C15—C14—C13	131.5 (2)	F8—C104—C103	120.6 (2)
N23—C14—C13	105.6 (2)	F8—C104—C105	119.5 (2)
N23—C14—C15	122.9 (2)	C103—C104—C105	119.9 (2)
C14—C15—C151	117.8 (2)	F9—C105—C104	119.2 (2)
C16—C15—C14	121.8 (2)	F9—C105—C106	120.8 (2)
C16—C15—C151	120.4 (2)	C106—C105—C104	119.9 (2)
C15—C16—C17	127.5 (2)	F10—C106—C101	119.4 (2)
N24—C16—C15	122.1 (2)	F10—C106—C105	118.6 (2)
N24—C16—C17	110.2 (2)	C105—C106—C101	122.0 (2)
C16—C17—Br7	127.80 (18)	C152—C151—C15	121.6 (2)
C18—C17—Br7	125.52 (19)	C156—C151—C15	121.6 (2)
C18—C17—C16	106.3 (2)	C156—C151—C152	116.8 (2)
C17—C18—Br8	125.01 (18)	F11—C152—C151	120.2 (2)
C17—C18—C19	106.6 (2)	F11—C152—C153	118.1 (2)
C19—C18—Br8	127.61 (17)	C153—C152—C151	121.7 (2)
C20—C19—C18	126.2 (2)	F12—C153—C152	120.4 (2)
N24—C19—C18	110.6 (2)	F12—C153—C154	119.6 (2)
N24—C19—C20	123.0 (2)	C154—C153—C152	120.0 (2)
C1—C20—C19	123.4 (2)	F13—C154—C153	119.9 (2)
C1—C20—C201	119.6 (2)	F13—C154—C155	120.2 (2)
C19—C20—C201	117.0 (2)	C155—C154—C153	120.0 (2)
C1—N21—C4	112.39 (19)	F14—C155—C154	119.9 (2)
C1—N21—H21	123.8	F14—C155—C156	120.5 (2)
C4—N21—H21	123.8	C154—C155—C156	119.6 (2)
C6—N22—C9	106.16 (19)	F15—C156—C151	119.9 (2)
C11—N23—H23	124.1	F15—C156—C155	118.3 (2)
C14—N23—C11	111.9 (2)	C155—C156—C151	121.9 (2)
C14—N23—H23	124.1	C202—C201—C20	120.6 (2)
Cl1S—C1S—H	108.0	C202—C201—C206	116.6 (2)
Cl1S—C1S—Cl2S	111.8 (3)	C206—C201—C20	122.8 (2)
Cl1S—C1S—Cl3S	110.9 (3)	F16—C202—C201	119.9 (2)
Cl2S—C1S—H	108.0	F16—C202—C203	117.9 (3)
Cl2S—C1S—Cl3S	110.1 (3)	C203—C202—C201	122.2 (3)
Cl3S—C1S—H	108.0	F17—C203—C202	120.5 (3)
C19—N24—C16	105.90 (19)	F17—C203—C204	119.8 (3)
C52—C51—C5	122.6 (11)	C204—C203—C202	119.7 (3)
C52—C51—C56	120.0	F18—C204—C203	120.4 (3)
C56—C51—C5	117.4 (11)	F18—C204—C205	119.9 (3)
F1—C52—C51	119.4 (12)	C203—C204—C205	119.7 (3)
F1—C52—C53	120.5 (11)	F19—C205—C204	120.2 (3)
C53—C52—C51	120.0	F19—C205—C206	119.9 (3)
F2—C53—C52	118.4 (7)	C206—C205—C204	119.9 (3)
F2—C53—C54	121.6 (7)	F20—C206—C201	120.0 (2)
C52—C53—C54	120.0	F20—C206—C205	118.1 (3)
F3—C54—C53	118.9 (6)	C205—C206—C201	121.9 (3)
			
C1—C2—C3—Br2	−173.32 (18)	C18—C19—C20—C1	−158.9 (3)
C1—C2—C3—C4	0.3 (3)	C18—C19—C20—C201	22.2 (4)
C1—C20—C201—C202	−117.1 (3)	C18—C19—N24—C16	−6.5 (3)
C1—C20—C201—C206	64.9 (4)	F18—C204—C205—F19	−0.9 (5)
Br1—C2—C3—Br2	−0.6 (3)	F18—C204—C205—C206	179.6 (3)
Br1—C2—C3—C4	172.97 (18)	C19—C20—C201—C202	61.9 (3)
C2—C1—C20—C19	−169.3 (3)	C19—C20—C201—C206	−116.1 (3)
C2—C1—C20—C201	9.6 (4)	F19—C205—C206—F20	0.5 (4)
C2—C1—N21—C4	−5.1 (3)	F19—C205—C206—C201	−178.5 (3)
C2—C3—C4—C5	173.4 (3)	C20—C1—C2—Br1	14.5 (4)
C2—C3—C4—N21	−3.2 (3)	C20—C1—C2—C3	−173.1 (3)
Br2—C3—C4—C5	−13.4 (4)	C20—C1—N21—C4	171.1 (2)
Br2—C3—C4—N21	170.02 (19)	C20—C19—N24—C16	167.9 (2)
C3—C4—C5—C6	169.0 (3)	C20—C201—C202—F16	3.1 (4)
C3—C4—C5—C51	−9.0 (9)	C20—C201—C202—C203	−177.3 (3)
C3—C4—C5—C51B	−12.9 (8)	C20—C201—C206—F20	−2.4 (4)
C3—C4—N21—C1	5.2 (3)	C20—C201—C206—C205	176.7 (3)
Br3—C7—C8—Br4	−0.7 (3)	N21—C1—C2—Br1	−169.59 (18)
Br3—C7—C8—C9	−174.94 (18)	N21—C1—C2—C3	2.8 (3)
C4—C5—C6—C7	165.5 (2)	N21—C1—C20—C19	15.5 (4)
C4—C5—C6—N22	−21.1 (4)	N21—C1—C20—C201	−165.6 (2)
C4—C5—C51—C52	−68.3 (13)	N21—C4—C5—C6	−15.0 (4)
C4—C5—C51—C56	113.2 (9)	N21—C4—C5—C51	167.0 (8)
C4—C5—C51B—C52B	−69.1 (10)	N21—C4—C5—C51B	163.1 (7)
C4—C5—C51B—C56B	112.2 (8)	N22—C6—C7—Br3	169.45 (19)
Br4—C8—C9—C10	22.4 (4)	N22—C6—C7—C8	−2.7 (3)
Br4—C8—C9—N22	−167.51 (18)	N22—C9—C10—C11	22.5 (4)
C5—C4—N21—C1	−171.6 (2)	N22—C9—C10—C101	−153.4 (2)
C5—C6—C7—Br3	−16.5 (4)	N23—C11—C12—Br5	−168.58 (18)
C5—C6—C7—C8	171.4 (2)	N23—C11—C12—C13	2.6 (3)
C5—C6—N22—C9	−167.7 (2)	N23—C14—C15—C16	−25.9 (4)
C5—C51—C52—F1	3.7 (15)	N23—C14—C15—C151	155.8 (2)
C5—C51—C52—C53	−178.5 (18)	F4—C55—C56—F5	−4 (2)
C5—C51—C56—F5	−1.1 (18)	F4—C55—C56—C51	176.7 (13)
C5—C51—C56—C55	178.6 (17)	F3—C54—C55—F4	0.9 (14)
C5—C51B—C52B—C53B	−178.7 (15)	F3—C54—C55—C56	177.8 (12)
C5—C51B—C52B—F1B	1.0 (13)	F2—C53—C54—F3	2.1 (13)
C5—C51B—C56B—C55B	178.6 (15)	F2—C53—C54—C55	179.9 (13)
C5—C51B—C56B—F5B	−1.6 (15)	F1—C52—C53—F2	−2.1 (17)
Br5—C12—C13—Br6	0.4 (3)	F1—C52—C53—C54	177.8 (17)
Br5—C12—C13—C14	171.48 (18)	N24—C16—C17—Br7	169.92 (17)
C6—C5—C51—C52	113.6 (10)	N24—C16—C17—C18	−3.0 (3)
C6—C5—C51—C56	−64.9 (12)	N24—C19—C20—C1	27.6 (4)
C6—C5—C51B—C52B	109.1 (8)	N24—C19—C20—C201	−151.4 (2)
C6—C5—C51B—C56B	−69.6 (11)	C51—C5—C6—C7	−16.5 (9)
C6—C7—C8—Br4	171.98 (18)	C51—C5—C6—N22	156.9 (8)
C6—C7—C8—C9	−2.2 (3)	C51—C52—C53—F2	−179.9 (12)
F6—C102—C103—F7	−0.4 (4)	C51—C52—C53—C54	0.0
F6—C102—C103—C104	178.5 (2)	C52—C51—C56—F5	−180 (2)
Br6—C13—C14—C15	−14.4 (4)	C52—C51—C56—C55	0.0
Br6—C13—C14—N23	168.31 (18)	C52—C53—C54—F3	−177.8 (12)
C7—C6—N22—C9	6.7 (3)	C52—C53—C54—C55	0.0
C7—C8—C9—C10	−163.6 (2)	C53—C54—C55—F4	−176.9 (12)
C7—C8—C9—N22	6.5 (3)	C53—C54—C55—C56	0.0
F7—C103—C104—F8	1.7 (4)	C54—C55—C56—F5	180 (2)
F7—C103—C104—C105	−179.5 (2)	C54—C55—C56—C51	0.0
Br7—C17—C18—Br8	−3.3 (3)	C56—C51—C52—F1	−177.8 (17)
Br7—C17—C18—C19	−174.12 (17)	C56—C51—C52—C53	0.0
C8—C9—C10—C11	−168.6 (2)	C51B—C5—C6—C7	−12.6 (8)
C8—C9—C10—C101	15.5 (4)	C51B—C5—C6—N22	160.8 (7)
C8—C9—N22—C6	−8.1 (3)	C51B—C52B—C53B—C54B	0.0
F8—C104—C105—F9	−1.0 (4)	C51B—C52B—C53B—F2B	179.5 (10)
F8—C104—C105—C106	178.2 (2)	C52B—C51B—C56B—C55B	0.0
Br8—C18—C19—C20	20.1 (4)	C52B—C51B—C56B—F5B	179.8 (17)
Br8—C18—C19—N24	−165.68 (18)	C52B—C53B—C54B—C55B	0.0
C9—C10—C11—C12	−158.5 (3)	C52B—C53B—C54B—F3B	−178.1 (10)
C9—C10—C11—N23	25.2 (4)	C53B—C54B—C55B—C56B	0.0
C9—C10—C101—C102	54.8 (3)	C53B—C54B—C55B—F4B	−179.3 (11)
C9—C10—C101—C106	−125.1 (3)	C54B—C55B—C56B—C51B	0.0
F9—C105—C106—F10	−2.3 (4)	C54B—C55B—C56B—F5B	−179.8 (17)
F9—C105—C106—C101	178.6 (2)	C56B—C51B—C52B—C53B	0.0
C10—C9—N22—C6	162.4 (2)	C56B—C51B—C52B—F1B	179.6 (15)
C10—C11—C12—Br5	14.6 (4)	F1B—C52B—C53B—C54B	−179.7 (14)
C10—C11—C12—C13	−174.2 (3)	F1B—C52B—C53B—F2B	−0.2 (14)
C10—C11—N23—C14	172.7 (2)	F2B—C53B—C54B—C55B	−179.5 (10)
C10—C101—C102—F6	0.5 (4)	F2B—C53B—C54B—F3B	2.4 (11)
C10—C101—C102—C103	−179.3 (2)	F3B—C54B—C55B—C56B	178.1 (10)
C10—C101—C106—F10	1.5 (4)	F3B—C54B—C55B—F4B	−1.2 (12)
C10—C101—C106—C105	−179.5 (2)	F4B—C55B—C56B—C51B	179.3 (11)
C11—C10—C101—C102	−121.3 (3)	F4B—C55B—C56B—F5B	−0.5 (16)
C11—C10—C101—C106	58.8 (3)	C101—C10—C11—C12	17.4 (4)
C11—C12—C13—Br6	−171.15 (18)	C101—C10—C11—N23	−158.9 (2)
C11—C12—C13—C14	0.0 (3)	C101—C102—C103—F7	179.5 (2)
F11—C152—C153—F12	2.7 (4)	C101—C102—C103—C104	−1.7 (4)
F11—C152—C153—C154	−179.0 (2)	C102—C101—C106—F10	−178.4 (2)
C12—C11—N23—C14	−4.4 (3)	C102—C101—C106—C105	0.6 (4)
C12—C13—C14—C15	174.7 (3)	C102—C103—C104—F8	−177.1 (2)
C12—C13—C14—N23	−2.6 (3)	C102—C103—C104—C105	1.7 (4)
F12—C153—C154—F13	−0.6 (4)	C103—C104—C105—F9	−179.8 (2)
F12—C153—C154—C155	178.9 (3)	C103—C104—C105—C106	−0.5 (4)
C13—C14—C15—C16	157.2 (3)	C104—C105—C106—F10	178.4 (2)
C13—C14—C15—C151	−21.1 (4)	C104—C105—C106—C101	−0.6 (4)
C13—C14—N23—C11	4.4 (3)	C106—C101—C102—F6	−179.6 (2)
F13—C154—C155—F14	0.1 (4)	C106—C101—C102—C103	0.6 (4)
F13—C154—C155—C156	178.1 (2)	C151—C15—C16—C17	−18.9 (4)
C14—C15—C16—C17	162.8 (2)	C151—C15—C16—N24	154.5 (2)
C14—C15—C16—N24	−23.7 (4)	C151—C152—C153—F12	−177.4 (2)
C14—C15—C151—C152	−53.8 (3)	C151—C152—C153—C154	0.8 (4)
C14—C15—C151—C156	125.3 (3)	C152—C151—C156—F15	−179.7 (2)
F14—C155—C156—F15	−0.9 (4)	C152—C151—C156—C155	0.6 (4)
F14—C155—C156—C151	178.8 (2)	C152—C153—C154—F13	−178.9 (3)
C15—C14—N23—C11	−173.2 (2)	C152—C153—C154—C155	0.6 (4)
C15—C16—C17—Br7	−16.0 (4)	C153—C154—C155—F14	−179.4 (2)
C15—C16—C17—C18	171.1 (2)	C153—C154—C155—C156	−1.3 (4)
C15—C16—N24—C19	−168.6 (2)	C154—C155—C156—F15	−178.9 (2)
C15—C151—C152—F11	−2.5 (4)	C154—C155—C156—C151	0.7 (4)
C15—C151—C152—C153	177.7 (2)	C156—C151—C152—F11	178.4 (2)
C15—C151—C156—F15	1.2 (4)	C156—C151—C152—C153	−1.4 (4)
C15—C151—C156—C155	−178.5 (2)	C201—C202—C203—F17	−179.5 (3)
C16—C15—C151—C152	127.9 (3)	C201—C202—C203—C204	0.1 (5)
C16—C15—C151—C156	−53.1 (3)	C202—C201—C206—F20	179.6 (2)
C16—C17—C18—Br8	169.77 (17)	C202—C201—C206—C205	−1.4 (4)
C16—C17—C18—C19	−1.0 (3)	C202—C203—C204—F18	179.9 (3)
F16—C202—C203—F17	0.2 (4)	C202—C203—C204—C205	−0.5 (5)
F16—C202—C203—C204	179.8 (3)	C203—C204—C205—F19	179.5 (3)
C17—C16—N24—C19	5.9 (3)	C203—C204—C205—C206	−0.1 (5)
C17—C18—C19—C20	−169.4 (2)	C204—C205—C206—F20	−179.9 (3)
C17—C18—C19—N24	4.8 (3)	C204—C205—C206—C201	1.1 (5)
F17—C203—C204—F18	−0.5 (5)	C206—C201—C202—F16	−178.8 (2)
F17—C203—C204—C205	179.1 (3)	C206—C201—C202—C203	0.8 (4)

### 2,3,7,8,12,13,17,18-Octabromo-5,10,15,20-tetrakis(pentafluorophenyl)porphyrin chloroform monosolvate (1_CHCl3) Hydrogen-bond geometry (Å, º)

**Table:**

*D*—H···*A*	*D*—H	H···*A*	*D*···*A*	*D*—H···*A*
N21—H21···N22	0.88	2.34	2.866 (3)	119
N21—H21···N24	0.88	2.42	2.928 (3)	117
N23—H23···N22	0.88	2.48	2.931 (3)	113
N23—H23···N24	0.88	2.46	2.895 (3)	111

### 2,3,7,8,12,13,17,18-Octabromo-5,10,15,20-tetrakis(pentafluorophenyl)porphyrin tetrahydrofuran monosolvate (1_THF) Crystal data

**Table:**

C_44_H_2_Br_8_F_20_N_4_·C_4_H_8_O	*F*(000) = 3176
*M**_r_* = 1677.88	*D*_x_ = 2.185 Mg m^−^^3^
Monoclinic, *P*2_1_/*n*	Mo *K*α radiation, λ = 0.71073 Å
*a* = 21.524 (9) Å	Cell parameters from 9873 reflections
*b* = 9.545 (4) Å	θ = 2.3–28.3°
*c* = 26.020 (11) Å	µ = 6.41 mm^−^^1^
β = 107.426 (7)°	*T* = 100 K
*V* = 5101 (4) Å^3^	Block fragment, blue
*Z* = 4	0.42 × 0.11 × 0.09 mm

### 2,3,7,8,12,13,17,18-Octabromo-5,10,15,20-tetrakis(pentafluorophenyl)porphyrin tetrahydrofuran monosolvate (1_THF) Data collection

**Table:**

Bruker APEXII CCD diffractometer	12689 independent reflections
Radiation source: microfocus sealed X-ray tube, graphite-monochromated	10079 reflections with *I* > 2σ(*I*)
Mirror optics monochromator	*R*_int_ = 0.082
Detector resolution: 7.9 pixels mm^-1^	θ_max_ = 28.3°, θ_min_ = 1.5°
φ and ω scans	*h* = −28→28
Absorption correction: multi-scan (SADABS; Bruker, 2016)	*k* = −12→12
*T*_min_ = 0.455, *T*_max_ = 0.746	*l* = −34→34
148293 measured reflections	

### 2,3,7,8,12,13,17,18-Octabromo-5,10,15,20-tetrakis(pentafluorophenyl)porphyrin tetrahydrofuran monosolvate (1_THF) Refinement

**Table:**

Refinement on *F*^2^	Primary atom site location: dual
Least-squares matrix: full	Hydrogen site location: mixed
*R*[*F*^2^ > 2σ(*F*^2^)] = 0.054	H atoms treated by a mixture of independent and constrained refinement
*wR*(*F*^2^) = 0.095	*w* = 1/[σ^2^(*F*_o_^2^) + 36.9739*P*] where *P* = (*F*_o_^2^ + 2*F*_c_^2^)/3
*S* = 1.21	(Δ/σ)_max_ = 0.001
12689 reflections	Δρ_max_ = 1.63 e Å^−^^3^
908 parameters	Δρ_min_ = −0.98 e Å^−^^3^
718 restraints	

### 2,3,7,8,12,13,17,18-Octabromo-5,10,15,20-tetrakis(pentafluorophenyl)porphyrin tetrahydrofuran monosolvate (1_THF) Special details

**Table:**

Geometry. All esds (except the esd in the dihedral angle between two l.s. planes) are estimated using the full covariance matrix. The cell esds are taken into account individually in the estimation of esds in distances, angles and torsion angles; correlations between esds in cell parameters are only used when they are defined by crystal symmetry. An approximate (isotropic) treatment of cell esds is used for estimating esds involving l.s. planes.
Refinement. Two perfluoro rings were modelled as disordered over two locations with restraints (SADI, SIMU) and constraints (EXYZ, EADP for ipso carbons C12/C12a and C21/C21a) with occupancies of 75:25 and 70:30%. Pyrrole hydrogens located on the difference map and refined using restraints (DFIX). There is a short intermolecular HL..HL contact Br5···Br5 of 3.23 Angstroms (symmetry code = 1-x,-y,1-z = 3_656). See ALERT B

### 2,3,7,8,12,13,17,18-Octabromo-5,10,15,20-tetrakis(pentafluorophenyl)porphyrin tetrahydrofuran monosolvate (1_THF) Fractional atomic coordinates and isotropic or equivalent isotropic displacement parameters (Å^2^)

**Table:**

	*x*	*y*	*z*	*U*_iso_*/*U*_eq_	Occ. (<1)
Br1	0.52232 (2)	0.99320 (5)	0.17627 (2)	0.01967 (11)	
Br2	0.65964 (2)	0.99488 (5)	0.28686 (2)	0.01897 (11)	
Br3	0.82447 (2)	0.44087 (5)	0.35875 (2)	0.01795 (11)	
Br4	0.77566 (3)	0.15494 (6)	0.41730 (2)	0.02278 (12)	
Br5	0.49322 (3)	0.11413 (6)	0.45284 (2)	0.02566 (13)	
Br6	0.35528 (3)	0.08167 (6)	0.34382 (2)	0.02386 (12)	
Br7	0.35141 (3)	0.15311 (6)	0.10022 (2)	0.02311 (12)	
Br8	0.39813 (3)	0.44804 (6)	0.04830 (2)	0.02922 (14)	
F1B	0.3598 (14)	0.762 (2)	0.1217 (9)	0.024 (3)	0.306 (17)
F2B	0.3219 (10)	0.893 (2)	0.0271 (8)	0.034 (3)	0.306 (17)
F3B	0.4084 (10)	0.924 (2)	−0.0300 (7)	0.037 (3)	0.306 (17)
F4B	0.5320 (10)	0.819 (2)	0.0078 (7)	0.033 (3)	0.306 (17)
F5B	0.5669 (13)	0.674 (3)	0.1029 (10)	0.022 (3)	0.306 (17)
F6	0.73871 (14)	0.7001 (3)	0.23590 (11)	0.0205 (7)	
F7	0.85729 (15)	0.8205 (3)	0.25703 (12)	0.0241 (7)	
F8	0.92243 (15)	0.9035 (4)	0.35882 (14)	0.0313 (8)	
F9	0.86678 (15)	0.8738 (3)	0.43919 (12)	0.0255 (7)	
F10	0.75021 (15)	0.7465 (3)	0.41955 (11)	0.0222 (7)	
F11B	0.6146 (14)	−0.081 (4)	0.3942 (12)	0.025 (3)	0.252 (18)
F12B	0.6698 (11)	−0.231 (3)	0.4832 (9)	0.030 (3)	0.252 (18)
F13B	0.7274 (11)	−0.093 (3)	0.5776 (9)	0.032 (3)	0.252 (18)
F14B	0.7159 (12)	0.188 (3)	0.5826 (10)	0.033 (3)	0.252 (18)
F15B	0.6587 (12)	0.339 (3)	0.4930 (12)	0.028 (3)	0.252 (18)
F16	0.40450 (15)	−0.0892 (3)	0.23781 (13)	0.0266 (7)	
F17	0.28883 (18)	−0.2265 (4)	0.20717 (15)	0.0360 (9)	
F18	0.17533 (17)	−0.0789 (4)	0.17611 (17)	0.0441 (10)	
F19	0.17774 (15)	0.2053 (4)	0.17564 (16)	0.0395 (9)	
F20	0.29175 (15)	0.3428 (3)	0.20559 (13)	0.0261 (7)	
N21	0.57239 (19)	0.6076 (4)	0.25003 (16)	0.0150 (9)	
N22	0.63177 (19)	0.4305 (4)	0.34616 (16)	0.0153 (9)	
H22	0.5882 (12)	0.470 (5)	0.339 (2)	0.015 (14)*	
N23	0.5165 (2)	0.2459 (4)	0.30937 (16)	0.0159 (9)	
N24	0.4668 (2)	0.4152 (5)	0.21131 (16)	0.0163 (9)	
H24	0.492 (2)	0.442 (6)	0.2476 (10)	0.032 (17)*	
C1	0.4597 (2)	0.4950 (6)	0.16626 (19)	0.0154 (10)	
C2	0.4854 (2)	0.6308 (5)	0.1660 (2)	0.0170 (10)	
C3	0.5348 (2)	0.6877 (5)	0.2093 (2)	0.0164 (10)	
C4	0.5587 (2)	0.8328 (5)	0.21547 (19)	0.0147 (10)	
C5	0.6130 (2)	0.8340 (5)	0.2584 (2)	0.0150 (10)	
C6	0.6230 (2)	0.6905 (5)	0.27870 (19)	0.0148 (10)	
C7	0.6794 (2)	0.6364 (5)	0.31605 (19)	0.0149 (10)	
C8	0.6838 (2)	0.5035 (5)	0.33991 (19)	0.0154 (10)	
C9	0.7404 (2)	0.4180 (5)	0.36414 (19)	0.0143 (10)	
C10	0.7210 (2)	0.3030 (5)	0.3861 (2)	0.0154 (10)	
C11	0.6514 (2)	0.3098 (5)	0.37521 (19)	0.0158 (10)	
C12	0.6088 (3)	0.2197 (5)	0.3909 (2)	0.0181 (11)	
C12A	0.6362 (3)	0.1253 (6)	0.4380 (2)	0.0225 (9)	0.252 (18)
C12F	0.6624 (10)	0.1997 (13)	0.4855 (4)	0.0246 (15)	0.252 (18)
C12E	0.6914 (12)	0.129 (3)	0.5333 (3)	0.0259 (16)	0.252 (18)
C12D	0.6943 (11)	−0.017 (3)	0.5336 (6)	0.0258 (16)	0.252 (18)
C12C	0.6680 (13)	−0.0913 (13)	0.4861 (9)	0.0250 (16)	0.252 (18)
C12B	0.6390 (10)	−0.0202 (6)	0.4383 (6)	0.0234 (15)	0.252 (18)
C13	0.5419 (3)	0.2086 (5)	0.3621 (2)	0.0176 (10)	
C14	0.4905 (2)	0.1467 (6)	0.38137 (18)	0.0166 (10)	
C15	0.4364 (2)	0.1415 (6)	0.3390 (2)	0.0190 (11)	
C16	0.4536 (2)	0.2014 (5)	0.29310 (19)	0.0145 (10)	
C17	0.4142 (2)	0.2103 (5)	0.2392 (2)	0.0151 (10)	
C18	0.4276 (2)	0.2986 (5)	0.2003 (2)	0.0159 (10)	
C19	0.3984 (2)	0.2980 (5)	0.14292 (19)	0.0160 (10)	
C20	0.4176 (2)	0.4162 (6)	0.12187 (19)	0.0175 (10)	
C21B	0.4602 (3)	0.7128 (6)	0.1155 (2)	0.0194 (9)	0.306 (17)
C22B	0.4009 (18)	0.775 (4)	0.0944 (13)	0.0222 (16)	0.306 (17)
C23B	0.3830 (13)	0.846 (3)	0.0440 (11)	0.0258 (16)	0.306 (17)
C24B	0.4304 (15)	0.854 (3)	0.0173 (9)	0.0268 (16)	0.306 (17)
C25B	0.4895 (12)	0.802 (3)	0.0375 (11)	0.0259 (16)	0.306 (17)
C26B	0.5064 (16)	0.729 (4)	0.0845 (15)	0.0226 (15)	0.306 (17)
C71	0.7412 (2)	0.7171 (5)	0.3271 (2)	0.0149 (10)	
C72	0.7695 (2)	0.7413 (5)	0.28649 (19)	0.0159 (10)	
C73	0.8303 (2)	0.8037 (5)	0.2964 (2)	0.0175 (10)	
C74	0.8631 (2)	0.8463 (5)	0.3483 (2)	0.0195 (11)	
C75	0.8353 (2)	0.8286 (5)	0.3890 (2)	0.0176 (10)	
C76	0.7754 (2)	0.7640 (5)	0.3785 (2)	0.0177 (10)	
C171	0.3519 (2)	0.1309 (6)	0.22187 (19)	0.0165 (10)	
C172	0.3489 (3)	−0.0143 (6)	0.2218 (2)	0.0210 (11)	
C173	0.2904 (3)	−0.0867 (6)	0.2065 (2)	0.0271 (13)	
C174	0.2327 (3)	−0.0110 (7)	0.1905 (3)	0.0315 (14)	
C175	0.2339 (3)	0.1325 (6)	0.1905 (2)	0.0252 (12)	
C176	0.2929 (3)	0.2012 (6)	0.2059 (2)	0.0207 (11)	
F1A	0.3574 (5)	0.7361 (10)	0.1329 (4)	0.0236 (17)	0.694 (17)
F2A	0.3068 (4)	0.8801 (10)	0.0399 (4)	0.0346 (19)	0.694 (17)
F3A	0.3808 (5)	0.9315 (9)	−0.0262 (3)	0.0399 (19)	0.694 (17)
F4A	0.5055 (5)	0.8370 (9)	0.0000 (3)	0.0370 (17)	0.694 (17)
F5A	0.5569 (6)	0.6956 (12)	0.0924 (4)	0.0267 (18)	0.694 (17)
F11A	0.6002 (5)	−0.0773 (12)	0.3832 (4)	0.0261 (16)	0.748 (18)
F12A	0.6504 (4)	−0.2514 (7)	0.4669 (4)	0.0321 (15)	0.748 (18)
F13A	0.7155 (4)	−0.1454 (10)	0.5655 (3)	0.0349 (16)	0.748 (18)
F14A	0.7237 (4)	0.1351 (10)	0.5815 (3)	0.0362 (16)	0.748 (18)
F15A	0.6742 (4)	0.3116 (9)	0.4979 (4)	0.0303 (16)	0.748 (18)
O25	0.5156 (2)	0.5565 (5)	0.3555 (2)	0.0452 (12)	
C26	0.4822 (3)	0.6868 (7)	0.3386 (3)	0.0428 (17)	
H26A	0.440036	0.670382	0.310628	0.051*	
H26B	0.508874	0.750268	0.323698	0.051*	
C27	0.4714 (3)	0.7495 (8)	0.3890 (3)	0.0485 (19)	
H27A	0.431987	0.809281	0.379803	0.058*	
H27B	0.509394	0.805839	0.409327	0.058*	
C28	0.4628 (4)	0.6212 (8)	0.4209 (3)	0.0492 (19)	
H28A	0.476700	0.640698	0.460102	0.059*	
H28B	0.417027	0.588821	0.409734	0.059*	
C29	0.5061 (5)	0.5173 (9)	0.4068 (4)	0.073 (3)	
H29A	0.548620	0.514797	0.435283	0.088*	
H29B	0.486356	0.422913	0.403966	0.088*	
C21A	0.4602 (3)	0.7128 (6)	0.1155 (2)	0.0194 (9)	0.694 (17)
C22A	0.3956 (8)	0.7599 (15)	0.1009 (5)	0.0214 (15)	0.694 (17)
C23A	0.3688 (5)	0.8339 (11)	0.0536 (4)	0.0242 (15)	0.694 (17)
C24A	0.4061 (6)	0.8611 (11)	0.0197 (4)	0.0271 (15)	0.694 (17)
C25A	0.4697 (6)	0.8157 (12)	0.0333 (4)	0.0258 (14)	0.694 (17)
C26A	0.4955 (6)	0.7428 (16)	0.0809 (6)	0.0227 (14)	0.694 (17)
C121	0.6362 (3)	0.1253 (6)	0.4380 (2)	0.0225 (9)	0.748 (18)
C122	0.6306 (4)	−0.0197 (8)	0.4319 (3)	0.0222 (11)	0.748 (18)
C123	0.6566 (5)	−0.1134 (9)	0.4740 (4)	0.0253 (12)	0.748 (18)
C124	0.6892 (4)	−0.0585 (11)	0.5241 (4)	0.0253 (13)	0.748 (18)
C125	0.6943 (5)	0.0823 (12)	0.5328 (3)	0.0255 (13)	0.748 (18)
C126	0.6682 (4)	0.1736 (10)	0.4902 (3)	0.0240 (12)	0.748 (18)

### 2,3,7,8,12,13,17,18-Octabromo-5,10,15,20-tetrakis(pentafluorophenyl)porphyrin tetrahydrofuran monosolvate (1_THF) Atomic displacement parameters (Å^2^)

**Table:**

	*U*^11^	*U*^22^	*U*^33^	*U*^12^	*U*^13^	*U*^23^
Br1	0.0205 (2)	0.0128 (2)	0.0221 (3)	0.0018 (2)	0.00074 (19)	0.0039 (2)
Br2	0.0225 (2)	0.0116 (2)	0.0198 (2)	−0.0023 (2)	0.00176 (19)	−0.0003 (2)
Br3	0.0163 (2)	0.0166 (2)	0.0195 (2)	0.0006 (2)	0.00298 (19)	−0.0003 (2)
Br4	0.0204 (3)	0.0165 (3)	0.0274 (3)	0.0033 (2)	0.0011 (2)	0.0070 (2)
Br5	0.0309 (3)	0.0302 (3)	0.0177 (3)	−0.0009 (2)	0.0100 (2)	0.0056 (2)
Br6	0.0197 (3)	0.0276 (3)	0.0268 (3)	−0.0009 (2)	0.0107 (2)	0.0072 (2)
Br7	0.0259 (3)	0.0183 (3)	0.0216 (3)	−0.0048 (2)	0.0017 (2)	−0.0021 (2)
Br8	0.0433 (3)	0.0249 (3)	0.0145 (2)	−0.0085 (3)	0.0011 (2)	0.0009 (2)
F1B	0.024 (4)	0.021 (5)	0.022 (5)	0.004 (4)	−0.001 (5)	0.008 (4)
F2B	0.037 (5)	0.022 (4)	0.029 (5)	0.001 (4)	−0.012 (4)	0.006 (4)
F3B	0.048 (6)	0.028 (5)	0.022 (4)	−0.002 (6)	−0.008 (5)	0.009 (4)
F4B	0.044 (6)	0.032 (5)	0.021 (5)	−0.007 (5)	0.007 (5)	0.010 (4)
F5B	0.027 (6)	0.022 (6)	0.018 (6)	−0.001 (5)	0.008 (5)	0.011 (5)
F6	0.0234 (16)	0.0227 (17)	0.0141 (14)	−0.0004 (13)	0.0034 (12)	−0.0023 (12)
F7	0.0283 (17)	0.0228 (17)	0.0242 (16)	−0.0036 (14)	0.0126 (13)	0.0019 (14)
F8	0.0222 (16)	0.033 (2)	0.0364 (19)	−0.0134 (15)	0.0055 (14)	−0.0040 (16)
F9	0.0270 (17)	0.0245 (18)	0.0193 (15)	−0.0054 (14)	−0.0019 (13)	−0.0059 (13)
F10	0.0260 (16)	0.0264 (17)	0.0151 (15)	−0.0026 (14)	0.0073 (12)	−0.0020 (13)
F11B	0.022 (6)	0.025 (5)	0.026 (6)	0.000 (5)	0.002 (5)	0.010 (5)
F12B	0.030 (5)	0.030 (5)	0.031 (5)	0.008 (5)	0.008 (4)	0.015 (5)
F13B	0.028 (5)	0.039 (6)	0.028 (5)	0.013 (5)	0.008 (4)	0.019 (5)
F14B	0.034 (5)	0.041 (6)	0.019 (5)	0.003 (6)	−0.001 (4)	0.008 (6)
F15B	0.025 (6)	0.031 (6)	0.024 (5)	0.002 (5)	−0.001 (5)	0.003 (5)
F16	0.0236 (16)	0.0167 (17)	0.0383 (19)	0.0016 (13)	0.0075 (14)	0.0057 (14)
F17	0.040 (2)	0.0193 (18)	0.049 (2)	−0.0109 (16)	0.0139 (18)	−0.0005 (16)
F18	0.0234 (18)	0.040 (2)	0.064 (3)	−0.0194 (17)	0.0056 (17)	−0.002 (2)
F19	0.0142 (16)	0.042 (2)	0.058 (2)	0.0033 (15)	0.0050 (16)	0.0043 (19)
F20	0.0218 (16)	0.0203 (17)	0.0354 (19)	0.0020 (13)	0.0074 (14)	0.0020 (14)
N21	0.015 (2)	0.014 (2)	0.014 (2)	−0.0024 (17)	0.0015 (16)	0.0022 (17)
N22	0.013 (2)	0.013 (2)	0.018 (2)	−0.0050 (17)	0.0025 (16)	0.0034 (17)
N23	0.016 (2)	0.016 (2)	0.014 (2)	−0.0011 (17)	0.0011 (16)	0.0026 (17)
N24	0.018 (2)	0.018 (2)	0.0109 (19)	−0.0022 (18)	0.0011 (16)	0.0020 (17)
C1	0.015 (2)	0.016 (2)	0.014 (2)	0.004 (2)	0.0034 (18)	0.003 (2)
C2	0.017 (2)	0.018 (3)	0.014 (2)	0.003 (2)	0.0029 (19)	0.003 (2)
C3	0.015 (2)	0.016 (3)	0.017 (2)	−0.001 (2)	0.0032 (19)	0.004 (2)
C4	0.015 (2)	0.013 (2)	0.017 (2)	0.0019 (19)	0.0045 (19)	0.003 (2)
C5	0.018 (2)	0.009 (2)	0.017 (2)	0.0016 (19)	0.0039 (19)	0.0008 (19)
C6	0.015 (2)	0.016 (3)	0.014 (2)	0.0005 (19)	0.0052 (19)	0.0027 (19)
C7	0.019 (2)	0.011 (2)	0.013 (2)	0.0016 (19)	0.0036 (19)	−0.0010 (19)
C8	0.017 (2)	0.015 (2)	0.012 (2)	−0.002 (2)	0.0022 (18)	0.000 (2)
C9	0.015 (2)	0.012 (2)	0.014 (2)	0.0000 (19)	0.0006 (18)	−0.0020 (19)
C10	0.017 (2)	0.011 (2)	0.016 (2)	0.0024 (19)	0.0005 (19)	0.0025 (19)
C11	0.020 (2)	0.013 (2)	0.012 (2)	0.000 (2)	0.0015 (19)	0.0008 (19)
C12	0.024 (3)	0.015 (3)	0.014 (2)	−0.001 (2)	0.004 (2)	0.002 (2)
C12A	0.0202 (17)	0.0260 (18)	0.0209 (17)	−0.0001 (16)	0.0055 (15)	0.0079 (16)
C12F	0.023 (3)	0.028 (3)	0.021 (2)	0.000 (3)	0.004 (2)	0.009 (2)
C12E	0.025 (2)	0.030 (3)	0.021 (2)	0.001 (3)	0.005 (2)	0.010 (3)
C12D	0.024 (2)	0.030 (3)	0.023 (3)	0.004 (3)	0.007 (2)	0.011 (3)
C12C	0.023 (3)	0.027 (3)	0.025 (3)	0.004 (2)	0.008 (2)	0.010 (3)
C12B	0.021 (3)	0.026 (3)	0.023 (3)	0.002 (2)	0.007 (2)	0.008 (2)
C13	0.024 (3)	0.012 (2)	0.016 (2)	−0.002 (2)	0.006 (2)	0.002 (2)
C14	0.024 (3)	0.019 (3)	0.007 (2)	0.000 (2)	0.0043 (19)	0.0037 (19)
C15	0.018 (2)	0.015 (3)	0.025 (3)	−0.003 (2)	0.008 (2)	0.002 (2)
C16	0.017 (2)	0.010 (2)	0.016 (2)	−0.0008 (19)	0.0046 (19)	0.0028 (19)
C17	0.013 (2)	0.015 (2)	0.017 (2)	0.0013 (19)	0.0045 (19)	0.002 (2)
C18	0.013 (2)	0.014 (2)	0.019 (2)	0.0030 (19)	0.0004 (19)	0.002 (2)
C19	0.016 (2)	0.018 (3)	0.014 (2)	0.000 (2)	0.0031 (19)	−0.001 (2)
C20	0.019 (2)	0.019 (3)	0.011 (2)	0.002 (2)	0.0000 (19)	0.002 (2)
C21B	0.0267 (19)	0.0146 (17)	0.0141 (17)	−0.0026 (16)	0.0018 (15)	0.0026 (15)
C22B	0.029 (3)	0.016 (3)	0.017 (3)	−0.001 (2)	0.000 (2)	0.003 (2)
C23B	0.032 (3)	0.019 (2)	0.019 (2)	−0.002 (2)	−0.003 (2)	0.005 (2)
C24B	0.035 (3)	0.020 (2)	0.019 (2)	−0.003 (3)	−0.001 (3)	0.005 (2)
C25B	0.034 (3)	0.021 (3)	0.019 (2)	−0.003 (3)	0.001 (3)	0.005 (2)
C26B	0.030 (3)	0.018 (3)	0.017 (2)	−0.003 (3)	0.002 (3)	0.005 (2)
C71	0.018 (2)	0.006 (2)	0.017 (2)	−0.0018 (19)	0.0010 (19)	0.0012 (19)
C72	0.020 (2)	0.011 (2)	0.014 (2)	0.002 (2)	0.0007 (19)	−0.0003 (19)
C73	0.020 (3)	0.012 (2)	0.022 (3)	0.001 (2)	0.009 (2)	0.003 (2)
C74	0.016 (2)	0.010 (2)	0.029 (3)	−0.003 (2)	0.003 (2)	−0.001 (2)
C75	0.019 (2)	0.013 (2)	0.017 (2)	−0.002 (2)	−0.001 (2)	−0.003 (2)
C76	0.021 (3)	0.013 (3)	0.019 (3)	0.003 (2)	0.005 (2)	0.000 (2)
C171	0.019 (2)	0.018 (3)	0.013 (2)	−0.002 (2)	0.0061 (19)	0.001 (2)
C172	0.020 (3)	0.019 (3)	0.024 (3)	−0.001 (2)	0.006 (2)	0.003 (2)
C173	0.030 (3)	0.020 (3)	0.032 (3)	−0.011 (2)	0.011 (3)	−0.004 (2)
C174	0.019 (3)	0.035 (4)	0.038 (3)	−0.014 (3)	0.005 (2)	0.000 (3)
C175	0.017 (3)	0.028 (3)	0.030 (3)	−0.001 (2)	0.006 (2)	0.002 (3)
C176	0.022 (3)	0.020 (3)	0.020 (3)	−0.003 (2)	0.006 (2)	0.000 (2)
F1A	0.022 (2)	0.021 (4)	0.027 (4)	0.004 (3)	0.006 (3)	0.009 (3)
F2A	0.030 (4)	0.027 (3)	0.033 (4)	0.002 (3)	−0.010 (3)	0.008 (3)
F3A	0.051 (4)	0.032 (3)	0.023 (3)	−0.001 (3)	−0.010 (3)	0.013 (2)
F4A	0.048 (4)	0.038 (3)	0.024 (3)	−0.008 (3)	0.010 (3)	0.008 (2)
F5A	0.031 (4)	0.029 (4)	0.022 (4)	−0.001 (3)	0.011 (3)	0.009 (3)
F11A	0.028 (4)	0.022 (2)	0.025 (4)	−0.002 (3)	0.003 (3)	0.002 (3)
F12A	0.036 (4)	0.022 (3)	0.041 (4)	0.006 (2)	0.015 (3)	0.015 (3)
F13A	0.033 (3)	0.041 (4)	0.030 (3)	0.010 (3)	0.009 (2)	0.020 (3)
F14A	0.036 (3)	0.044 (4)	0.021 (2)	−0.003 (3)	−0.003 (2)	0.008 (3)
F15A	0.034 (4)	0.028 (3)	0.021 (3)	−0.002 (3)	−0.004 (3)	0.003 (2)
O25	0.044 (3)	0.029 (3)	0.072 (4)	0.013 (2)	0.031 (3)	0.010 (2)
C26	0.031 (4)	0.029 (4)	0.068 (5)	0.009 (3)	0.015 (3)	0.007 (3)
C27	0.032 (4)	0.040 (4)	0.075 (5)	−0.001 (3)	0.018 (4)	−0.012 (4)
C28	0.051 (5)	0.042 (4)	0.055 (5)	0.012 (4)	0.017 (4)	−0.001 (4)
C29	0.112 (8)	0.040 (5)	0.090 (7)	0.039 (5)	0.064 (6)	0.023 (5)
C21A	0.0267 (19)	0.0146 (17)	0.0141 (17)	−0.0026 (16)	0.0018 (15)	0.0026 (15)
C22A	0.028 (3)	0.016 (2)	0.016 (3)	−0.002 (2)	0.000 (2)	0.003 (2)
C23A	0.031 (3)	0.017 (2)	0.018 (2)	−0.001 (2)	−0.003 (2)	0.003 (2)
C24A	0.035 (3)	0.019 (2)	0.020 (2)	−0.003 (2)	−0.003 (2)	0.005 (2)
C25A	0.034 (3)	0.021 (2)	0.019 (2)	−0.004 (2)	0.001 (2)	0.005 (2)
C26A	0.029 (3)	0.018 (2)	0.018 (2)	−0.003 (2)	0.002 (2)	0.003 (2)
C121	0.0202 (17)	0.0260 (18)	0.0209 (17)	−0.0001 (16)	0.0055 (15)	0.0079 (16)
C122	0.020 (2)	0.025 (2)	0.022 (2)	0.0027 (19)	0.0075 (19)	0.009 (2)
C123	0.023 (2)	0.027 (2)	0.027 (2)	0.004 (2)	0.009 (2)	0.009 (2)
C124	0.023 (2)	0.030 (3)	0.023 (2)	0.005 (2)	0.0083 (19)	0.012 (2)
C125	0.024 (2)	0.030 (3)	0.021 (2)	0.000 (2)	0.0046 (18)	0.009 (2)
C126	0.023 (2)	0.027 (2)	0.021 (2)	0.000 (2)	0.0042 (18)	0.008 (2)

### 2,3,7,8,12,13,17,18-Octabromo-5,10,15,20-tetrakis(pentafluorophenyl)porphyrin tetrahydrofuran monosolvate (1_THF) Geometric parameters (Å, º)

**Table:**

Br1—C4	1.876 (5)	C13—C14	1.468 (7)
Br2—C5	1.862 (5)	C14—C15	1.343 (7)
Br3—C9	1.869 (5)	C15—C16	1.468 (7)
Br4—C10	1.863 (5)	C16—C17	1.408 (7)
Br5—C14	1.869 (5)	C17—C18	1.411 (7)
Br6—C15	1.877 (5)	C17—C171	1.488 (7)
Br7—C19	1.870 (5)	C18—C19	1.437 (7)
Br8—C20	1.858 (5)	C19—C20	1.370 (7)
F1B—C22B	1.30 (4)	C21B—C22B	1.37 (4)
F2B—C23B	1.33 (3)	C21B—C26B	1.46 (4)
F3B—C24B	1.35 (3)	C22B—C23B	1.42 (3)
F4B—C25B	1.37 (3)	C23B—C24B	1.40 (3)
F5B—C26B	1.35 (3)	C24B—C25B	1.32 (3)
F6—C72	1.343 (5)	C25B—C26B	1.36 (4)
F7—C73	1.329 (6)	C71—C72	1.389 (7)
F8—C74	1.339 (6)	C71—C76	1.392 (7)
F9—C75	1.350 (5)	C72—C73	1.389 (7)
F10—C76	1.346 (6)	C73—C74	1.386 (7)
F11B—C12B	1.25 (3)	C74—C75	1.373 (8)
F12B—C12C	1.34 (2)	C75—C76	1.380 (7)
F13B—C12D	1.36 (2)	C171—C172	1.388 (7)
F14B—C12E	1.36 (3)	C171—C176	1.385 (7)
F15B—C12F	1.35 (3)	C172—C173	1.385 (7)
F16—C172	1.349 (6)	C173—C174	1.390 (9)
F17—C173	1.335 (7)	C174—C175	1.370 (9)
F18—C174	1.344 (6)	C175—C176	1.378 (7)
F19—C175	1.346 (6)	F1A—C22A	1.354 (15)
F20—C176	1.352 (6)	F2A—C23A	1.348 (12)
N21—C3	1.358 (6)	F3A—C24A	1.334 (12)
N21—C6	1.371 (6)	F4A—C25A	1.337 (13)
N22—H22	0.974 (10)	F5A—C26A	1.344 (15)
N22—C8	1.369 (6)	F11A—C122	1.356 (12)
N22—C11	1.373 (6)	F12A—C123	1.331 (11)
N23—C13	1.363 (6)	F13A—C124	1.343 (9)
N23—C16	1.361 (6)	F14A—C125	1.334 (11)
N24—H24	0.975 (10)	F15A—C126	1.333 (11)
N24—C1	1.368 (6)	O25—C26	1.439 (8)
N24—C18	1.374 (6)	O25—C29	1.459 (9)
C1—C2	1.410 (7)	C26—H26A	0.9900
C1—C20	1.448 (7)	C26—H26B	0.9900
C2—C3	1.407 (7)	C26—C27	1.521 (10)
C2—C21B	1.487 (7)	C27—H27A	0.9900
C2—C21A	1.487 (7)	C27—H27B	0.9900
C3—C4	1.469 (7)	C27—C28	1.522 (11)
C4—C5	1.354 (7)	C28—H28A	0.9900
C5—C6	1.461 (7)	C28—H28B	0.9900
C6—C7	1.407 (7)	C28—C29	1.481 (10)
C7—C8	1.403 (7)	C29—H29A	0.9900
C7—C71	1.490 (7)	C29—H29B	0.9900
C8—C9	1.446 (7)	C21A—C22A	1.400 (18)
C9—C10	1.361 (7)	C21A—C26A	1.370 (19)
C10—C11	1.440 (7)	C22A—C23A	1.385 (15)
C11—C12	1.404 (7)	C23A—C24A	1.385 (14)
C12—C12A	1.493 (7)	C24A—C25A	1.377 (13)
C12—C13	1.414 (7)	C25A—C26A	1.384 (16)
C12—C121	1.493 (7)	C121—C122	1.395 (11)
C12A—C12F	1.3900	C121—C126	1.405 (10)
C12A—C12B	1.3900	C122—C123	1.396 (10)
C12F—C12E	1.3900	C123—C124	1.385 (11)
C12E—C12D	1.3900	C124—C125	1.362 (12)
C12D—C12C	1.3900	C125—C126	1.389 (11)
C12C—C12B	1.3900		
			
C3—N21—C6	106.8 (4)	C24B—C25B—C26B	121 (3)
C8—N22—H22	124 (3)	C26B—C25B—F4B	122 (2)
C8—N22—C11	111.4 (4)	F5B—C26B—C21B	120 (3)
C11—N22—H22	123 (3)	F5B—C26B—C25B	119 (3)
C16—N23—C13	107.0 (4)	C25B—C26B—C21B	120 (2)
C1—N24—H24	125 (4)	C72—C71—C7	120.6 (4)
C1—N24—C18	111.7 (4)	C72—C71—C76	116.8 (5)
C18—N24—H24	123 (4)	C76—C71—C7	122.5 (5)
N24—C1—C2	124.9 (5)	F6—C72—C71	119.8 (4)
N24—C1—C20	106.1 (4)	F6—C72—C73	117.9 (4)
C2—C1—C20	128.9 (5)	C73—C72—C71	122.3 (5)
C1—C2—C21B	116.6 (4)	F7—C73—C72	120.9 (5)
C1—C2—C21A	116.6 (4)	F7—C73—C74	120.2 (5)
C3—C2—C1	123.7 (5)	C74—C73—C72	118.9 (5)
C3—C2—C21B	119.6 (5)	F8—C74—C73	119.7 (5)
C3—C2—C21A	119.6 (5)	F8—C74—C75	120.2 (5)
N21—C3—C2	122.5 (5)	C75—C74—C73	120.2 (5)
N21—C3—C4	109.8 (4)	F9—C75—C74	119.8 (4)
C2—C3—C4	127.3 (5)	F9—C75—C76	120.2 (5)
C3—C4—Br1	129.5 (4)	C74—C75—C76	119.9 (5)
C5—C4—Br1	123.9 (4)	F10—C76—C71	120.0 (5)
C5—C4—C3	106.6 (4)	F10—C76—C75	118.2 (4)
C4—C5—Br2	124.4 (4)	C75—C76—C71	121.9 (5)
C4—C5—C6	106.7 (4)	C172—C171—C17	123.1 (5)
C6—C5—Br2	128.8 (4)	C176—C171—C17	120.4 (5)
N21—C6—C5	109.7 (4)	C176—C171—C172	116.5 (5)
N21—C6—C7	123.0 (5)	F16—C172—C171	119.5 (5)
C7—C6—C5	126.7 (5)	F16—C172—C173	118.0 (5)
C6—C7—C71	118.7 (4)	C173—C172—C171	122.4 (5)
C8—C7—C6	124.6 (5)	F17—C173—C172	121.3 (5)
C8—C7—C71	116.3 (4)	F17—C173—C174	120.0 (5)
N22—C8—C7	124.3 (5)	C172—C173—C174	118.7 (5)
N22—C8—C9	105.9 (4)	F18—C174—C173	119.8 (6)
C7—C8—C9	129.8 (5)	F18—C174—C175	119.9 (6)
C8—C9—Br3	127.6 (4)	C175—C174—C173	120.3 (5)
C10—C9—Br3	123.6 (4)	F19—C175—C174	120.0 (5)
C10—C9—C8	108.2 (4)	F19—C175—C176	120.5 (5)
C9—C10—Br4	124.1 (4)	C174—C175—C176	119.5 (5)
C9—C10—C11	108.3 (4)	F20—C176—C171	120.0 (5)
C11—C10—Br4	127.2 (4)	F20—C176—C175	117.4 (5)
N22—C11—C10	106.0 (4)	C175—C176—C171	122.6 (5)
N22—C11—C12	123.7 (5)	C26—O25—C29	107.9 (5)
C12—C11—C10	130.3 (5)	O25—C26—H26A	110.6
C11—C12—C12A	118.4 (5)	O25—C26—H26B	110.6
C11—C12—C13	122.8 (5)	O25—C26—C27	105.5 (6)
C11—C12—C121	118.4 (5)	H26A—C26—H26B	108.8
C13—C12—C12A	118.6 (5)	C27—C26—H26A	110.6
C13—C12—C121	118.6 (5)	C27—C26—H26B	110.6
C12F—C12A—C12	112.2 (7)	C26—C27—H27A	111.1
C12F—C12A—C12B	120.0	C26—C27—H27B	111.1
C12B—C12A—C12	127.8 (7)	C26—C27—C28	103.3 (6)
F15B—C12F—C12A	127.2 (14)	H27A—C27—H27B	109.1
F15B—C12F—C12E	112.5 (14)	C28—C27—H27A	111.1
C12E—C12F—C12A	120.0	C28—C27—H27B	111.1
F14B—C12E—C12F	125.8 (14)	C27—C28—H28A	111.3
F14B—C12E—C12D	114.2 (14)	C27—C28—H28B	111.3
C12F—C12E—C12D	120.0	H28A—C28—H28B	109.2
F13B—C12D—C12E	123.0 (15)	C29—C28—C27	102.4 (6)
F13B—C12D—C12C	116.7 (15)	C29—C28—H28A	111.3
C12E—C12D—C12C	120.0	C29—C28—H28B	111.3
F12B—C12C—C12D	123.2 (14)	O25—C29—C28	109.1 (6)
F12B—C12C—C12B	116.8 (14)	O25—C29—H29A	109.9
C12B—C12C—C12D	120.0	O25—C29—H29B	109.9
F11B—C12B—C12A	116.8 (18)	C28—C29—H29A	109.9
F11B—C12B—C12C	123.2 (18)	C28—C29—H29B	109.9
C12C—C12B—C12A	120.0	H29A—C29—H29B	108.3
N23—C13—C12	123.4 (5)	C22A—C21A—C2	118.6 (6)
N23—C13—C14	109.5 (4)	C26A—C21A—C2	124.3 (6)
C12—C13—C14	126.9 (5)	C26A—C21A—C22A	117.1 (7)
C13—C14—Br5	127.2 (4)	F1A—C22A—C21A	121.1 (9)
C15—C14—Br5	124.8 (4)	F1A—C22A—C23A	117.6 (13)
C15—C14—C13	106.9 (4)	C23A—C22A—C21A	121.3 (10)
C14—C15—Br6	123.5 (4)	F2A—C23A—C22A	121.3 (10)
C14—C15—C16	106.6 (4)	F2A—C23A—C24A	119.1 (8)
C16—C15—Br6	129.7 (4)	C24A—C23A—C22A	119.7 (10)
N23—C16—C15	109.8 (4)	F3A—C24A—C23A	120.4 (9)
N23—C16—C17	122.5 (4)	F3A—C24A—C25A	119.7 (10)
C17—C16—C15	127.7 (4)	C25A—C24A—C23A	119.9 (9)
C16—C17—C18	123.9 (5)	F4A—C25A—C24A	120.5 (10)
C16—C17—C171	119.4 (4)	F4A—C25A—C26A	120.1 (10)
C18—C17—C171	116.6 (4)	C24A—C25A—C26A	119.4 (10)
N24—C18—C17	125.5 (5)	F5A—C26A—C21A	119.1 (10)
N24—C18—C19	105.6 (4)	F5A—C26A—C25A	118.1 (13)
C17—C18—C19	128.5 (5)	C21A—C26A—C25A	122.7 (10)
C18—C19—Br7	127.7 (4)	C122—C121—C12	120.3 (6)
C20—C19—Br7	123.0 (4)	C122—C121—C126	115.9 (6)
C20—C19—C18	108.8 (4)	C126—C121—C12	123.7 (6)
C1—C20—Br8	129.1 (4)	F11A—C122—C121	120.7 (8)
C19—C20—Br8	123.0 (4)	F11A—C122—C123	116.2 (8)
C19—C20—C1	107.5 (4)	C121—C122—C123	123.1 (8)
C22B—C21B—C2	129.4 (13)	F12A—C123—C122	121.7 (8)
C22B—C21B—C26B	116.1 (17)	F12A—C123—C124	120.4 (7)
C26B—C21B—C2	114.5 (12)	C124—C123—C122	117.9 (8)
F1B—C22B—C21B	116 (2)	F13A—C124—C123	119.6 (8)
F1B—C22B—C23B	121 (3)	F13A—C124—C125	118.9 (8)
C21B—C22B—C23B	123 (3)	C125—C124—C123	121.5 (7)
F2B—C23B—C22B	116 (2)	F14A—C125—C124	121.5 (7)
F2B—C23B—C24B	128 (2)	F14A—C125—C126	118.9 (8)
C24B—C23B—C22B	116 (2)	C124—C125—C126	119.6 (7)
F3B—C24B—C23B	112 (2)	F15A—C126—C121	117.8 (7)
C25B—C24B—F3B	125 (2)	F15A—C126—C125	120.1 (8)
C25B—C24B—C23B	123 (2)	C125—C126—C121	122.0 (8)
C24B—C25B—F4B	117 (2)		
			
Br1—C4—C5—Br2	1.5 (7)	C12A—C12—C13—N23	−154.4 (5)
Br1—C4—C5—C6	177.4 (3)	C12A—C12—C13—C14	20.2 (8)
Br2—C5—C6—N21	171.6 (4)	C12A—C12F—C12E—F14B	−177 (3)
Br2—C5—C6—C7	−17.4 (8)	C12A—C12F—C12E—C12D	0.0
Br3—C9—C10—Br4	3.2 (6)	C12F—C12A—C12B—F11B	−180 (2)
Br3—C9—C10—C11	−170.2 (3)	C12F—C12A—C12B—C12C	0.0
Br4—C10—C11—N22	−172.6 (4)	C12F—C12E—C12D—F13B	173 (2)
Br4—C10—C11—C12	9.5 (8)	C12F—C12E—C12D—C12C	0.0
Br5—C14—C15—Br6	−6.7 (7)	C12E—C12D—C12C—F12B	179 (3)
Br5—C14—C15—C16	168.6 (4)	C12E—C12D—C12C—C12B	0.0
Br6—C15—C16—N23	172.5 (4)	C12D—C12C—C12B—F11B	180 (3)
Br6—C15—C16—C17	−9.6 (8)	C12D—C12C—C12B—C12A	0.0
Br7—C19—C20—Br8	1.0 (6)	C12B—C12A—C12F—F15B	−174 (3)
Br7—C19—C20—C1	−172.6 (4)	C12B—C12A—C12F—C12E	0.0
F1B—C22B—C23B—F2B	−2 (4)	C13—N23—C16—C15	4.3 (6)
F1B—C22B—C23B—C24B	−179 (3)	C13—N23—C16—C17	−173.7 (5)
F2B—C23B—C24B—F3B	3 (4)	C13—C12—C12A—C12F	−120.0 (10)
F2B—C23B—C24B—C25B	−179 (3)	C13—C12—C12A—C12B	61.6 (13)
F3B—C24B—C25B—F4B	0 (4)	C13—C12—C121—C122	56.6 (9)
F3B—C24B—C25B—C26B	−178 (3)	C13—C12—C121—C126	−122.9 (7)
F4B—C25B—C26B—F5B	1 (4)	C13—C14—C15—Br6	−175.7 (4)
F4B—C25B—C26B—C21B	180 (2)	C13—C14—C15—C16	−0.5 (6)
F6—C72—C73—F7	−0.8 (7)	C14—C15—C16—N23	−2.4 (6)
F6—C72—C73—C74	−179.6 (4)	C14—C15—C16—C17	175.6 (5)
F7—C73—C74—F8	−0.5 (7)	C15—C16—C17—C18	163.5 (5)
F7—C73—C74—C75	−179.8 (5)	C15—C16—C17—C171	−11.8 (8)
F8—C74—C75—F9	2.5 (8)	C16—N23—C13—C12	170.8 (5)
F8—C74—C75—C76	−177.2 (5)	C16—N23—C13—C14	−4.6 (6)
F9—C75—C76—F10	−0.8 (7)	C16—C17—C18—N24	−20.9 (8)
F9—C75—C76—C71	179.4 (5)	C16—C17—C18—C19	167.4 (5)
F12B—C12C—C12B—F11B	1 (2)	C16—C17—C171—C172	−64.6 (7)
F12B—C12C—C12B—C12A	−179 (2)	C16—C17—C171—C176	113.9 (6)
F13B—C12D—C12C—F12B	5 (3)	C17—C18—C19—Br7	−18.5 (8)
F13B—C12D—C12C—C12B	−173.8 (19)	C17—C18—C19—C20	169.5 (5)
F14B—C12E—C12D—F13B	−10 (3)	C17—C171—C172—F16	−0.3 (8)
F14B—C12E—C12D—C12C	177 (2)	C17—C171—C172—C173	178.8 (5)
F15B—C12F—C12E—F14B	−2 (2)	C17—C171—C176—F20	0.5 (8)
F15B—C12F—C12E—C12D	175 (2)	C17—C171—C176—C175	−178.8 (5)
F16—C172—C173—F17	−0.4 (8)	C18—N24—C1—C2	169.7 (5)
F16—C172—C173—C174	179.2 (5)	C18—N24—C1—C20	−6.1 (6)
F17—C173—C174—F18	0.4 (9)	C18—C17—C171—C172	119.7 (6)
F17—C173—C174—C175	179.1 (6)	C18—C17—C171—C176	−61.8 (7)
F18—C174—C175—F19	−0.4 (9)	C18—C19—C20—Br8	173.3 (4)
F18—C174—C175—C176	179.2 (5)	C18—C19—C20—C1	−0.2 (6)
F19—C175—C176—F20	0.1 (8)	C20—C1—C2—C3	−168.4 (5)
F19—C175—C176—C171	179.5 (5)	C20—C1—C2—C21B	8.4 (8)
N21—C3—C4—Br1	−173.3 (4)	C20—C1—C2—C21A	8.4 (8)
N21—C3—C4—C5	3.7 (6)	C21B—C2—C3—N21	−161.3 (5)
N21—C6—C7—C8	−19.1 (8)	C21B—C2—C3—C4	11.7 (8)
N21—C6—C7—C71	153.7 (5)	C21B—C22B—C23B—F2B	175 (3)
N22—C8—C9—Br3	168.2 (4)	C21B—C22B—C23B—C24B	−2 (4)
N22—C8—C9—C10	−3.2 (5)	C22B—C21B—C26B—F5B	178 (3)
N22—C11—C12—C12A	−157.2 (5)	C22B—C21B—C26B—C25B	−1 (4)
N22—C11—C12—C13	26.5 (8)	C22B—C23B—C24B—F3B	−180 (2)
N22—C11—C12—C121	−157.2 (5)	C22B—C23B—C24B—C25B	−2 (4)
N23—C13—C14—Br5	−165.5 (4)	C23B—C24B—C25B—F4B	−178 (2)
N23—C13—C14—C15	3.2 (6)	C23B—C24B—C25B—C26B	4 (4)
N23—C16—C17—C18	−18.8 (8)	C24B—C25B—C26B—F5B	178 (3)
N23—C16—C17—C171	165.9 (5)	C24B—C25B—C26B—C21B	−3 (4)
N24—C1—C2—C3	16.8 (8)	C26B—C21B—C22B—F1B	−180 (3)
N24—C1—C2—C21B	−166.5 (5)	C26B—C21B—C22B—C23B	3 (4)
N24—C1—C2—C21A	−166.5 (5)	C71—C7—C8—N22	162.3 (5)
N24—C1—C20—Br8	−169.3 (4)	C71—C7—C8—C9	−15.1 (8)
N24—C1—C20—C19	3.8 (6)	C71—C72—C73—F7	177.3 (5)
N24—C18—C19—Br7	168.5 (4)	C71—C72—C73—C74	−1.6 (8)
N24—C18—C19—C20	−3.4 (6)	C72—C71—C76—F10	178.7 (4)
C1—N24—C18—C17	−167.2 (5)	C72—C71—C76—C75	−1.5 (7)
C1—N24—C18—C19	6.0 (6)	C72—C73—C74—F8	178.4 (5)
C1—C2—C3—N21	15.3 (8)	C72—C73—C74—C75	−0.9 (8)
C1—C2—C3—C4	−171.6 (5)	C73—C74—C75—F9	−178.2 (5)
C1—C2—C21B—C22B	73 (2)	C73—C74—C75—C76	2.1 (8)
C1—C2—C21B—C26B	−107.6 (17)	C74—C75—C76—F10	178.9 (5)
C1—C2—C21A—C22A	69.4 (9)	C74—C75—C76—C71	−0.9 (8)
C1—C2—C21A—C26A	−108.8 (10)	C76—C71—C72—F6	−179.2 (4)
C2—C1—C20—Br8	15.1 (8)	C76—C71—C72—C73	2.8 (7)
C2—C1—C20—C19	−171.9 (5)	C171—C17—C18—N24	154.5 (5)
C2—C3—C4—Br1	12.9 (8)	C171—C17—C18—C19	−17.1 (8)
C2—C3—C4—C5	−170.1 (5)	C171—C172—C173—F17	−179.5 (5)
C2—C21B—C22B—F1B	0 (4)	C171—C172—C173—C174	0.1 (9)
C2—C21B—C22B—C23B	−177.5 (18)	C172—C171—C176—F20	179.1 (5)
C2—C21B—C26B—F5B	−1 (4)	C172—C171—C176—C175	−0.2 (8)
C2—C21B—C26B—C25B	180 (2)	C172—C173—C174—F18	−179.2 (5)
C2—C21A—C22A—F1A	2.4 (16)	C172—C173—C174—C175	−0.5 (9)
C2—C21A—C22A—C23A	−178.5 (9)	C173—C174—C175—F19	−179.1 (5)
C2—C21A—C26A—F5A	0.4 (17)	C173—C174—C175—C176	0.6 (10)
C2—C21A—C26A—C25A	177.9 (10)	C174—C175—C176—F20	−179.5 (5)
C3—N21—C6—C5	6.2 (5)	C174—C175—C176—C171	−0.2 (9)
C3—N21—C6—C7	−165.2 (5)	C176—C171—C172—F16	−178.8 (4)
C3—C2—C21B—C22B	−110 (2)	C176—C171—C172—C173	0.2 (8)
C3—C2—C21B—C26B	69.2 (17)	F1A—C22A—C23A—F2A	−0.3 (17)
C3—C2—C21A—C22A	−113.8 (9)	F1A—C22A—C23A—C24A	179.8 (10)
C3—C2—C21A—C26A	68.0 (11)	F2A—C23A—C24A—F3A	−0.3 (14)
C3—C4—C5—Br2	−175.6 (4)	F2A—C23A—C24A—C25A	179.5 (10)
C3—C4—C5—C6	0.2 (6)	F3A—C24A—C25A—F4A	−2.5 (15)
C4—C5—C6—N21	−4.0 (6)	F3A—C24A—C25A—C26A	180.0 (11)
C4—C5—C6—C7	167.1 (5)	F4A—C25A—C26A—F5A	0.3 (19)
C5—C6—C7—C8	170.9 (5)	F4A—C25A—C26A—C21A	−177.2 (11)
C5—C6—C7—C71	−16.2 (8)	F11A—C122—C123—F12A	−1.5 (12)
C6—N21—C3—C2	168.1 (5)	F11A—C122—C123—C124	178.6 (9)
C6—N21—C3—C4	−6.1 (6)	F12A—C123—C124—F13A	0.9 (12)
C6—C7—C8—N22	−24.6 (8)	F12A—C123—C124—C125	−177.8 (9)
C6—C7—C8—C9	157.9 (5)	F13A—C124—C125—F14A	−1.1 (13)
C6—C7—C71—C72	−62.6 (7)	F13A—C124—C125—C126	179.0 (8)
C6—C7—C71—C76	121.4 (5)	F14A—C125—C126—F15A	1.8 (12)
C7—C8—C9—Br3	−14.0 (8)	F14A—C125—C126—C121	−179.6 (9)
C7—C8—C9—C10	174.6 (5)	O25—C26—C27—C28	−32.3 (7)
C7—C71—C72—F6	4.6 (7)	C26—O25—C29—C28	3.1 (10)
C7—C71—C72—C73	−173.4 (5)	C26—C27—C28—C29	33.1 (8)
C7—C71—C76—F10	−5.2 (7)	C27—C28—C29—O25	−23.0 (10)
C7—C71—C76—C75	174.6 (5)	C29—O25—C26—C27	18.4 (8)
C8—N22—C11—C10	−2.7 (6)	C21A—C2—C3—N21	−161.3 (5)
C8—N22—C11—C12	175.5 (5)	C21A—C2—C3—C4	11.7 (8)
C8—C7—C71—C72	110.8 (5)	C21A—C22A—C23A—F2A	−179.5 (10)
C8—C7—C71—C76	−65.1 (6)	C21A—C22A—C23A—C24A	0.6 (17)
C8—C9—C10—Br4	175.0 (4)	C22A—C21A—C26A—F5A	−177.8 (12)
C8—C9—C10—C11	1.7 (6)	C22A—C21A—C26A—C25A	−0.3 (18)
C9—C10—C11—N22	0.5 (6)	C22A—C23A—C24A—F3A	179.6 (10)
C9—C10—C11—C12	−177.5 (5)	C22A—C23A—C24A—C25A	−0.6 (15)
C10—C11—C12—C12A	20.4 (8)	C23A—C24A—C25A—F4A	177.7 (10)
C10—C11—C12—C13	−155.8 (5)	C23A—C24A—C25A—C26A	0.2 (16)
C10—C11—C12—C121	20.4 (8)	C24A—C25A—C26A—F5A	177.8 (11)
C11—N22—C8—C7	−174.3 (5)	C24A—C25A—C26A—C21A	0.3 (19)
C11—N22—C8—C9	3.6 (5)	C26A—C21A—C22A—F1A	−179.3 (12)
C11—C12—C12A—C12F	63.7 (11)	C26A—C21A—C22A—C23A	−0.2 (17)
C11—C12—C12A—C12B	−114.7 (12)	C121—C12—C13—N23	−154.4 (5)
C11—C12—C13—N23	21.8 (8)	C121—C12—C13—C14	20.2 (8)
C11—C12—C13—C14	−163.6 (5)	C121—C122—C123—F12A	−180.0 (9)
C11—C12—C121—C122	−119.7 (7)	C121—C122—C123—C124	0.2 (11)
C11—C12—C121—C126	60.7 (9)	C122—C121—C126—F15A	−179.5 (9)
C12—C12A—C12F—F15B	8 (3)	C122—C121—C126—C125	1.8 (11)
C12—C12A—C12F—C12E	−178.5 (7)	C122—C123—C124—F13A	−179.3 (8)
C12—C12A—C12B—F11B	−2 (3)	C122—C123—C124—C125	2.0 (12)
C12—C12A—C12B—C12C	178.3 (8)	C123—C124—C125—F14A	177.6 (9)
C12—C13—C14—Br5	19.3 (8)	C123—C124—C125—C126	−2.3 (12)
C12—C13—C14—C15	−172.1 (5)	C124—C125—C126—F15A	−178.3 (9)
C12—C121—C122—F11A	0.0 (12)	C124—C125—C126—C121	0.3 (12)
C12—C121—C122—C123	178.3 (6)	C126—C121—C122—F11A	179.6 (9)
C12—C121—C126—F15A	0.0 (12)	C126—C121—C122—C123	−2.1 (11)
C12—C121—C126—C125	−178.6 (6)		

### 2,3,7,8,12,13,17,18-Octabromo-5,10,15,20-tetrakis(pentafluorophenyl)porphyrin tetrahydrofuran monosolvate (1_THF) Hydrogen-bond geometry (Å, º)

**Table:**

*D*—H···*A*	*D*—H	H···*A*	*D*···*A*	*D*—H···*A*
N22—H22···O25	0.97 (1)	1.92 (2)	2.849 (6)	158 (5)
